# Review of Antimicrobial Nanocoatings in Medicine and Dentistry: Mechanisms of Action, Biocompatibility Performance, Safety, and Benefits Compared to Antibiotics

**DOI:** 10.1021/acsnano.2c12488

**Published:** 2023-04-07

**Authors:** James Butler, Richard D. Handy, Mathew Upton, Alexandros Besinis

**Affiliations:** † School of Engineering, Computing and Mathematics, Faculty of Science and Engineering, University of Plymouth, Drake Circus, Plymouth PL4 8AA, United Kingdom; ‡ School of Biological and Marine Sciences, Faculty of Science and Engineering, University of Plymouth, Drake Circus, Plymouth PL4 8AA, United Kingdom; § School of Biomedical Sciences, Faculty of Health, University of Plymouth, Drake Circus, Plymouth PL4 8AA, United Kingdom; ∥ Peninsula Dental School, Faculty of Health, University of Plymouth, Drake Circus, Plymouth PL4 8AA, United Kingdom

**Keywords:** antimicrobial, resistance, antibacterial, antibiofilm, antibiotics, nanoparticle, nanomaterial, nanocoating, surface, safety

## Abstract

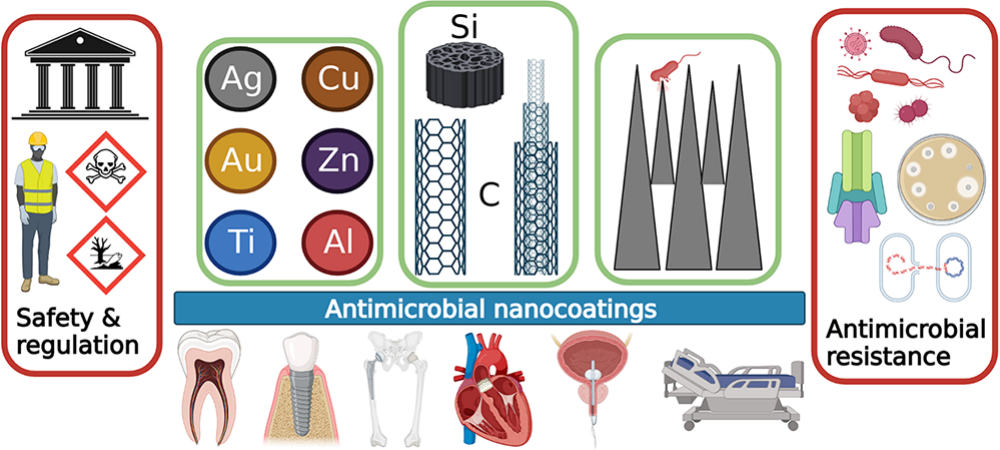

This review discusses topics relevant to the development of antimicrobial nanocoatings and nanoscale surface modifications for medical and dental applications. Nanomaterials have unique properties compared to their micro- and macro-scale counterparts and can be used to reduce or inhibit bacterial growth, surface colonization and biofilm development. Generally, nanocoatings exert their antimicrobial effects through biochemical reactions, production of reactive oxygen species or ionic release, while modified nanotopographies create a physically hostile surface for bacteria, killing cells via biomechanical damage. Nanocoatings may consist of metal nanoparticles including silver, copper, gold, zinc, titanium, and aluminum, while nonmetallic compounds used in nanocoatings may be carbon-based in the form of graphene or carbon nanotubes, or composed of silica or chitosan. Surface nanotopography can be modified by the inclusion of nanoprotrusions or black silicon. Two or more nanomaterials can be combined to form nanocomposites with distinct chemical or physical characteristics, allowing combination of different properties such as antimicrobial activity, biocompatibility, strength, and durability. Despite their wide range of applications in medical engineering, questions have been raised regarding potential toxicity and hazards. Current legal frameworks do not effectively regulate antimicrobial nanocoatings in matters of safety, with open questions remaining about risk analysis and occupational exposure limits not considering coating-based approaches. Bacterial resistance to nanomaterials is also a concern, especially where it may affect wider antimicrobial resistance. Nanocoatings have excellent potential for future use, but safe development of antimicrobials requires careful consideration of the “One Health” agenda, appropriate legislation, and risk assessment.

Engineered nanomaterials (ENMs) are clusters of atoms forming structures that have at least one dimension in the size range of 1–100 nm and can be found in different shapes and forms including nanoparticles, nanocrystals, nanorods and nanofibers.^1^ The behavior of ENMs can differ significantly from that of their bulk counterparts because their properties are not determined by their mass or chemical composition exclusively, as with most macro-materials. Certain factors affect the biological interactions of ENMs including their particle size,^2,3^ shape and surface area to volume ratio,^4,5^ crystallinity^6^ and surface charge.^7^ The unique properties and behaviors of nanomaterials in comparison to their micro- and macro-scale counterparts are the driving force behind the growing body of research in nanotechnology, which allows materials to be developed with specific desired properties. A range of ENMs have been found to have potent antimicrobial properties and as such have enormous potential in medical engineering applications where inhibition of bacterial growth and colonization is important. In recent years, the mechanisms of action of ENMs have become better understood and the exact effects that ENMs can have on bacterial or eukaryotic cells are finally being described, allowing optimization of their antimicrobial performance while maintaining biocompatibility and reducing ecological impact.

This review addresses the state of up-to-date research on the development, application and testing of ENMs with intrinsic antimicrobial properties as surface coatings for medical and dental applications. There are plentiful publications examining nanomaterials as antimicrobial agents^8−11^ or as carriers for antimicrobial drug delivery.^12,13^ However, this review discusses the use of ENMs in the form of antimicrobial surface nanocoatings and modification of the surface nanotopography to achieve infection prevention and control (IPC) in medicine and dentistry. The electronic search was conducted by applying a combination of subject terms and keywords on databases including PubMed, Scopus, Google Scholar, and Web of Science. The keywords applied to the searches were: (nanomaterial OR nanoparticle OR nanocoating OR nanotechnology) AND (antimicrobial OR antibacterial OR antifungal OR antibiofilm OR infection). Quality criteria included an assessment of experimental design and appropriate controls, comparisons, and conclusions in the published and peer-reviewed English-language literature. Publications with insufficient detail or relevance, poor descriptions of methodology, lack of replication, or inadequate material characterization were excluded. Examples were also chosen to show a representative selection of materials and applications; these selected examples from the published literature are presented in Tables 1–4. Representative example images of nanocoatings are also shown in Figures 2 and 4. The aims of this review are: (a) to present and discuss the types of antimicrobial nanocoatings available where the nanomaterial itself is intrinsically antimicrobial and assess their reported efficacy, (b) to evaluate the importance and relevance of nanocoatings and surface nanotopography as alternative antimicrobial strategies in the wider context of antimicrobial resistance and infection prevention and control, and (c) to discuss the general pitfalls and safety considerations associated with clinical applications.

**Table 1:** Summary of Additional Selected Examples from the Published Literature Regarding Application of Metal and Metal Oxide Nanoparticles as Antimicrobial Nanocoatings

Nanocoating description	Aim of application	Key methods^a^	Target organisms	Key results^a^	Source
Quaternary ammonium-modified gold nanoclusters	Orthodontic devices	48 h disc immersion in bacterial suspension	*Streptococcus mutans*	Reduced biofilm mass (85%) and viability (95%)	(129)
		CVSA and CLSM biofilm quantification		Good nanocoating stability	
		Biocompatibility for oral keratinocytes		No hemolysis and negligible cellular toxicity found	
Nickel–titanium wires coated with ZnO NPs by an electrochemical deposition method	Orthodontic wires	Agar diffusion test	*Streptococcus pyogenes,*	ZOIs observed against all bacteria investigated (mean 3.57–6.25 mm), with stronger activity against Gram positives (mean 4.25–6.25 mm)	(178)
			*Staphylococcus aureus,*		
			*Escherichia coli*		
ZnO NPs (12–27 nm) applied to TiZrNb alloy by plasma electrolytic oxidation	Dental and orthopedic implants	Sample immersion in bacterial suspension	*Staphylococcus aureus*	>2 log reduction in biofilm after 2 h	(179)
		Adherent cells enumerated		ZnO NPs-containing ceramic oxide coating hydrophobicity may affect eukaryotic cell attachment	
		Biocompatibility tested by seeding with U2OS cells and performing Alamar blue testing			
Ti or Ti–Zr surfaces were coated with a dual layer of ZnO nanospheres and nanorods synthesized by a hydrothermal method	Dental implants to prevent peri-implantitis	Sample immersion in bacterial suspension	*Escherichia coli, Staphylococcus aureus*	Rapid release from ZnO nanospheres with up to 2-fold higher short-term antibacterial activity	(180)
		Viable counts after 6 or 48 h		Slower release from ZnO nanorods with longer-term antibacterial properties	
		SEM to assess antibiofilm activity on surfaces coated with bacterial suspension after 2 h		30–70% *Staphylococcus aureus* biofilm inhibition after 6 h	
		*In vivo* activity in rats measured by implantation		*In vivo Staphylococcus aureus* inhibition (60–80%) over 2 weeks	
Ag NPs (38 nm) from silver nitrate applied to surgical nylon threads	Antimicrobial wound dressing material	Agar diffusion test with AgNO_3_ solution and sterile water controls	6 Gram positive bacteria, 4 Gram-negatives	>15 mm ZOIs against *Pseudomonas aeruginosa*, *Bacillus subtilis*, *Micrococcus luteus*, *Bacillus megaterium*, and *Staphylococcus aureus*	(181)
			3 molds	>12 mm ZOIs against *Rhizopus stolonifera* and *Candida albicans*	
			1 yeast		
Silver NPs deposited on both sides of cotton gauzes by technology based on *in situ* photoreduction of AgNO_3_	Application to prevent wound infections	Agar diffusion tests	*Staphylococcus aureus*	Growth and biofilm proliferation significantly reduced (3.5–4.5 log)	(182)
		Sample immersed in bacterial suspension and cells enumerated, CLSM and SEM		0.5% (*w/v*) had little effect on cell viability and high stability	
		Cytotoxicity on mouse embryonic fibroblasts and human keratinocytes with MTT assay		4% (*w/v*) showed 80% reduced cell viability and several fold higher silver release	
A “simultaneous sonochemical/enzymatic process” used to apply ZnO NPs to cotton	Medical textiles	Sample immersed in bacterial suspension and bacterial enumeration after 1 h	*Escherichia coli*, *Staphylococcus aureus*	Nonwashed samples were up to 98% more efficacious against *Escherichia coli*	(183)
		Durability evaluated after 10 wash cycles		Cellulase treatment improved durability of antimicrobial effect	
Coatings of ZnO (120–180 nm) or CuO (18–20 nm) NPs were applied to a teeth model	Antibiofilm coatings to improve oral health	Biofilm assays on artificial teeth	*Streptococcus mutans*	Biofilm formation inhibited by ZnO (85%) and CuO (70%)	(184)
		CVSA after 24 h		Effect was solely antibiofilm	
		Fixed teeth visualize with SEM			
TiO_2_ nanocoatings on Ti implants formed by an aqueous plasma electrodeposition technique	Photoactivated antimicrobial properties for titanium implants	TiO_2_-coated Ti specimen activated with infrared laser for 30 s	*Staphylococcus aureus*	5 min exposure reduced viability to 23%	(185)
		Samples immersed in bacterial suspension		30 min exposure reduced viability to 9%	
		Real time *in situ* imagining with live/dead-CLSM			

a CVSA: crystal violet solubilization assay; CLSM: confocal laser scanning microscopy; SEM: scanning electron microscopy; ZOI: zone of inhibition.

## Antimicrobial Mechanisms of Nanoparticles

**Figure 1:**
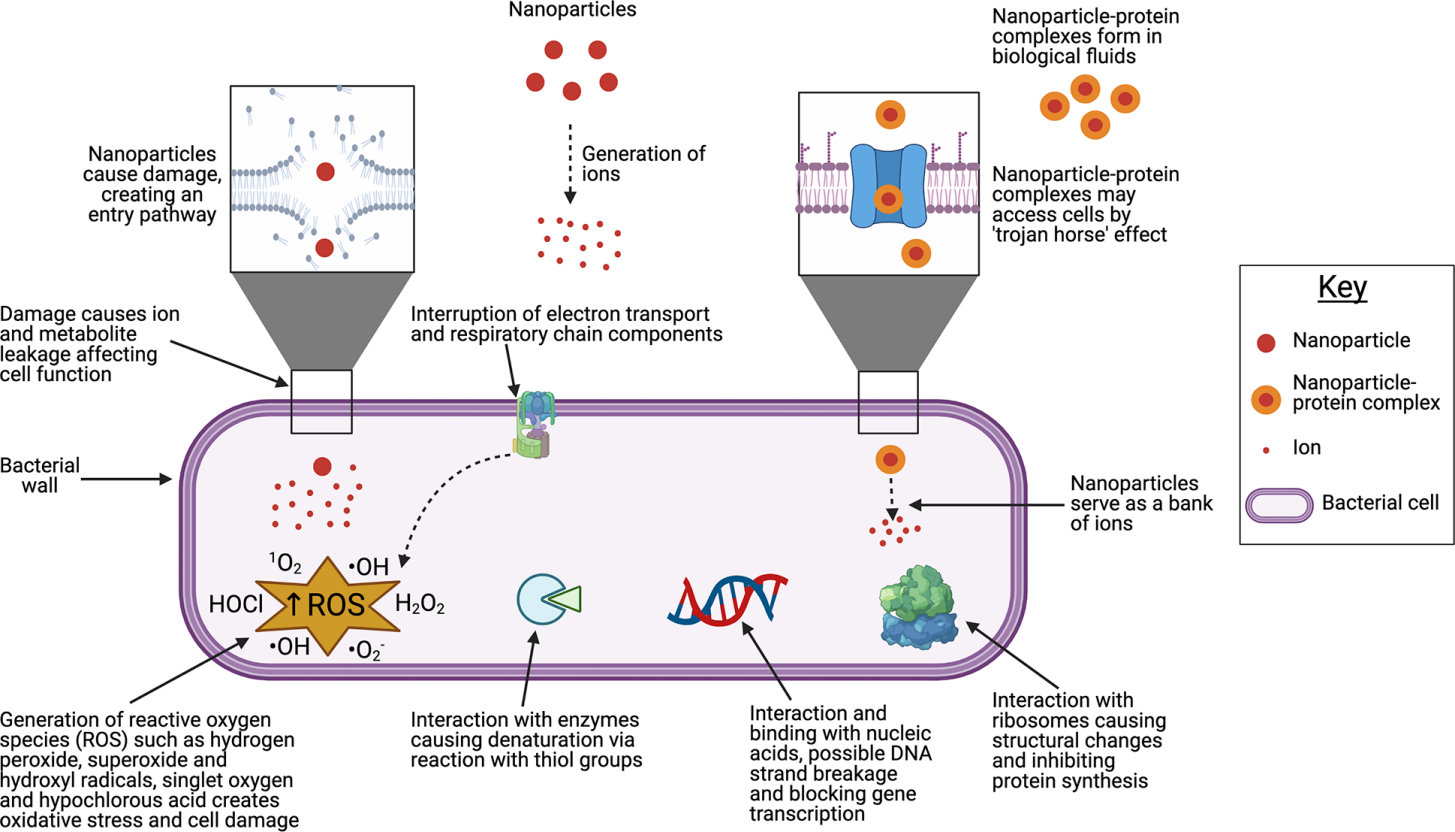
Antimicrobial mechanisms of nanoparticles. Nanoparticles gain entry by damaging the cell wall. Damage to the cell wall by nanoparticle entry may itself lead to leakage of ions and metabolites, conferring an antimicrobial effect. Nanoparticles act as a reservoir of ions which go on to interact intracellularly with ribosomes, nucleic acids, and enzymes, disrupting normal function. Interruption of the respiratory chain leads to generation of reactive oxygen species which create oxidative stress and damage cell components. Figure was created using BioRender.com.

The antimicrobial mechanisms of nanoparticles (NPs) are increasingly being understood in detail and are summarized in Figure 1. Generally, these mechanisms can be classified as direct contact-mediated killing and ion-mediated killing. Direct contact-mediated killing involves NP anchorage to and infiltration of the bacterial cell wall; this leads to membrane damage, leakage of cellular contents and ultimately may alone result in bacterial death.^14−17^ In this way, large NPs that cannot translocate the bacterial cell wall can still exert bactericidal effects by adsorption and thereby causing mechanical deformation leading to cell rupture and death.^18^ Upon penetration of the cell wall, NPs gain access to the cell interior and interfere with the function of intracellular biomolecules or structures such as proteins, organelles, and DNA either by direct interactions or by generation of ions. It is possible that NPs may also gain access to the cell without causing membrane damage by way of the protein corona effect, in which NPs in biological environments become modified by adsorption of biomolecules to their surface.^19,20^ Protein-occluded NPs become akin to a “trojan horse” and gain easier entry into the cell; however, while this has been shown to occur in eukaryotic cells,^21,22^ it is not known whether the mechanism can be generalized to include bacteria.

The generation of ions (*e.g.*, Ag^+^ or Cu^2+^) is broadly correlated with NP surface area, with greater surface area leading to greater ion production and thus greater antimicrobial activity.^23^ NPs may function as a “bank” of ions once inside the cell, continuously releasing them and prolonging or strengthening the antibacterial effect. Liberated metal cations can interact with thiol (sulfhydryl) groups in bacterial enzymes, forming stable bonds and disrupting function in essential molecules involved in transmembrane energy generation and electrolyte transport.^24^ Metal cations such as Ag^+^ can uncouple the respiratory electron transport chain from oxidative phosphorylation and interfere with penetration of H^+^ and phosphate into membranes.^25−27^ Within the bacterial cell, metal cations can form complexes with nuclear material by intercalation between base pairs, disrupting hydrogen bonds and ultimately preventing effective cell division.^28,29^ The production of reactive oxygen species (ROS), either by disruption of the thioredoxin system^30^ or by interaction with the respiratory chain and interruption of intracellular O_2_ reduction,^31^ is a major ion-mediated killing mechanism.

Generally, the mechanisms mentioned above overlap and cumulatively contribute to an antimicrobial effect. In some cases, however, certain mechanisms are considered to be more prominent for specific nanomaterials than for others. For example, nanosilver binds to the thiol groups of cysteine residues, which are frequently crucial for many proteins to maintain their integrity and function.^32^ Meanwhile, for nanomaterials consisting of oxides, such as TiO_2_, ZnO, CuO and Al_2_O_3_, toxicity to bacteria is predominantly the result of ROS generation.^33−35^ However, it is also clear that nonoxide NPs such as Se NPs^36^ and NPs composed of Ag, Cu, Fe, Mn, Co, Au, or Pt also generate ROS.^37^

## Biofilm Development and Antimicrobial Resistance

Biofilms are communities of bacteria organized into localized, heterogeneous and sessile aggregations that form when bacteria accumulate and adhere to surfaces, forming a thin but robust layer. The bacteria in biofilms are embedded within and secrete a mixture of biomolecules making up a dynamic matrix collectively termed extracellular polymeric substances (EPS). The EPS is composed of a complex assembly of protein, polysaccharide, and extracellular DNA, resulting in a three-dimensional architecture.^38,39^ The EPS has several roles including physical protection from shear forces, antimicrobials, and immune responses, enabling the diffusion of nutrients through the biofilm, and facilitating horizontal transfer of genes.^40,41^ The EPS layer confers a level of hydrophobicity which prevents permeation by most extraneous molecules and makes the biofilm very resilient, with some authors even referring to it as “omniphobic” – *i.e.*, repelling all substances.^42^ Experiments conducted *in vitro* have demonstrated that bacteria residing in mature biofilms can be between 10–1,000 fold more resistant to antibiotics than their equivalent planktonic cells, demonstrating the extremely robust nature of biofilms once formed.^43,44^ In this way, biofilm formation represents a major strategy allowing bacteria to defend against antimicrobial attack, facilitating resistance. Biofilms are ubiquitous in the environment, increasingly being considered the predominant means by which microbes thrive in their niche,^45^ including in the human body, but can present particular health concerns due to their ability to harbor pathogens and resist disinfectants^46,47^ and antibiotics.^48−51^

Antimicrobial resistance (AMR) is the outcome of microorganisms changing over time to be able to survive exposure to antimicrobial medicines such as antibiotics which are designed to kill them or inhibit their growth. The recent repeated warnings regarding the rise of AMR in bacteria, the major clinical challenges that this imposes,^52^ and the various national and international efforts to develop novel antimicrobials in order to maintain our ability to fight bacterial infections underscore the importance of antimicrobial nanomaterials to biomedical research, engineering, and clinical practice. One study found that the burden of antibiotic-resistant infections is comparable to the cumulative burden of influenza, tuberculosis, and HIV, most seriously affecting children aged <1 year and the elderly aged >65 years.^53^ Furthermore, it was reported that about 75% of the total antibiotic-resistant infection burden was associated with healthcare and 39% of all antibiotic-resistant infections are caused by bacteria with resistance to last-line or last-resort antibiotics, indicating that they are very difficult or even potentially impossible to treat. The UK government-commissioned O’Neill review^54^ on drug-resistant infections reported that at current rates, by 2050, AMR will lead to 10 million deaths a year, a 2.0–3.5% reduction in gross domestic product and will cost the world up to US$100 trillion. A study of the global AMR burden in 2019 estimated that 4.95 million deaths were associated with bacterial AMR, with 1.27 million deaths directly attributable to AMR.^55^ Unfortunately, the discovery and development of new antibiotics is not straightforward; no majorly impactful classes of antibiotics were introduced between 1962 and 2000,^56^ although the approval of daptomycin^57^ by the US Food and Drug Administration (FDA) in 2003 is often cited as one example of success. The global antibiotics market is dominated by classes introduced half a century ago^56^ and the majority of the pharmaceutical industry has dismantled or scaled back its antibiotic research laboratories, leaving an inadequate antibiotic pipeline and lack of industry infrastructure and expertise.^58,59^ The divestment in antibiotic R&D by the pharmaceutical industry is largely driven by poor returns on investment and it is now widely acknowledged that reimbursement for antibiotic development needs to be delinked from sales volumes.^60^ There are several international initiatives now in place that aim to “fix” the antibiotic R&D funding model: a UK scheme is being trialed where the Government will pay manufacturers a fixed fee for access to new antibiotics; similar approaches are being adopted in Germany and Sweden with a premium being paid for selected antibacterial agents; and in the US the PASTEUR Act will ensure annual revenues for new antibiotics meet a minimum level that is acceptable to industry.^61^

To address the recommendations of the O’Neill report, and ultimately reduce the global burden of AMR, both new antibiotics and new alternative antimicrobial strategies are urgently needed. As biofilms–once formed–provide such an effective barrier against antimicrobial attack, novel strategies which inhibit biofilm formation must be sought.

## Use of Nanocoatings as a Strategy for Infection Prevention and Control

**Figure 2:**
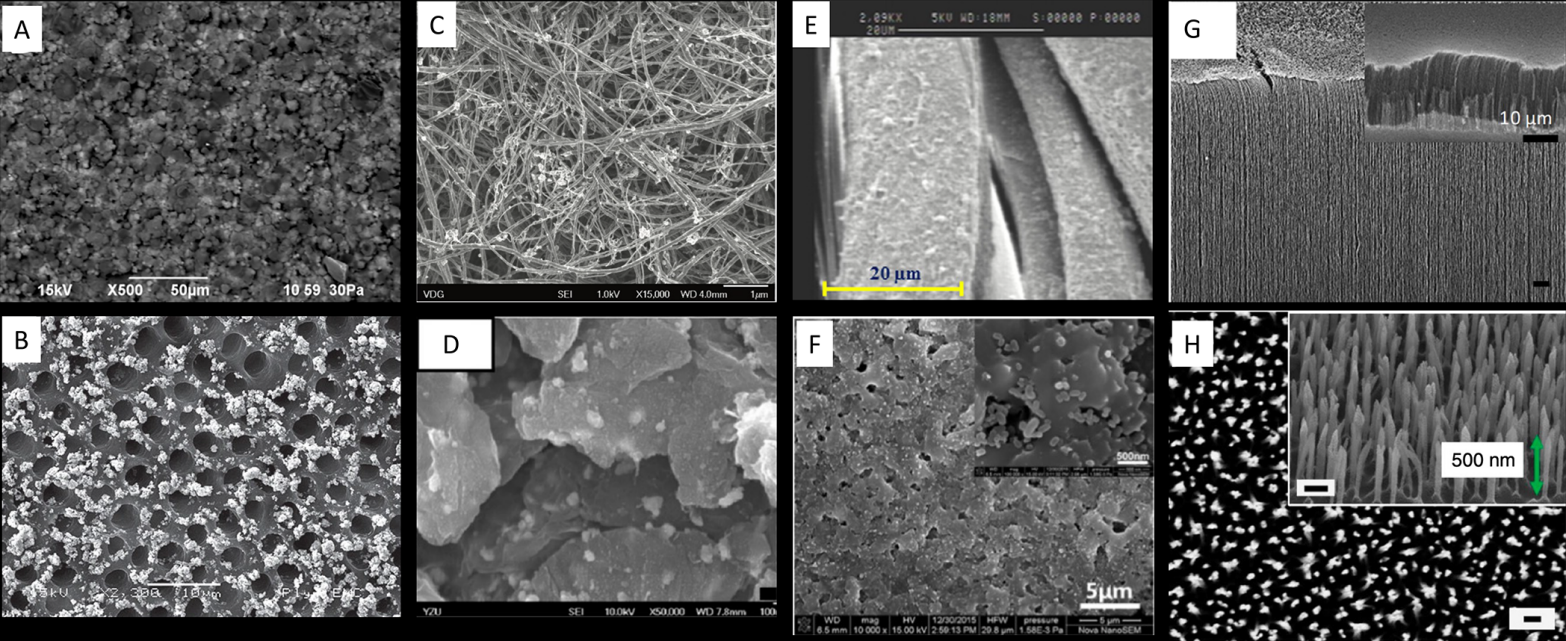
Representative scanning electron micrographs of antimicrobial nanocoatings. (A) Poly(methyl methacrylate) and silver nanoparticles desposited on silicon wafers. Reprinted with permission from ref (76). Copyright 2017 Elsevier. (B) Dentine coated with silver nanoparticles. Reprinted with permission from ref (77). Copyright 2014 Taylor & Francis Ltd. (C) Multiwalled carbon nanotubes decorated with silver nanoparticles. Reprinted with permission under a Creative Commons CC BY 3.0 License from ref (78). Copyright 2014 Hindawi Publishing Corporation. (D) Silver nanoparticles and zinc oxide nanoparticles embedded on graphene oxide. Reprinted with permission under a Creative Commons CC BY 4.0 License from ref (79). Copyright 2019 MDPI. (E) Fabric coated with poly(styrenesulfonate), chitosan and silver nanoparticles. Reprinted with permission from ref (80). Copyright 2020 Elsevier. (F) Silica nanoparticles applied to a titanium substrate by a microarc oxidation technique. Reprinted with permission from ref (81). Copyright 2017 Elsevier. (G) High aspect ratio (30 μm) vertically aligned carbon nanotubes. Reprinted with permission from ref (82). Copyright 2018 American Chemical Society. (H) Upper surface of black silicon with the green arrow indicating the relative height of the nanoprotrusion on the surface. Reprinted with permission from ref (83). Copyright 2013 Springer Nature.

As the effectiveness of currently available antibiotics is being undermined by rising AMR, nanotechnology seems to be a promising alternative strategy for treatment or IPC. Certain types of free NPs suspended in solutions have been found to be highly effective antimicrobials under *in vitro* conditions.^62−64^ However, their application in an immobilized form, such as nanocoatings, is a way to maximize their antibacterial efficacy while minimizing material loss (see representative examples in Figure 2). Regarding surface application of antimicrobials, the ideal scenario for IPC would be inhibition of initial biofilm formation, which requires interruption of bacterial adherence to substrates or early toxicity to bacteria. A “race for the surface” effect has been suggested, in which the first cells colonizing a surface tend to be the ones to successfully develop a community on that surface.^65,66^ An experimental setup investigating the “race for the surface” between eukaryotic U2OS osteosarcoma cells and *Staphylococcus epidermidis* demonstrated realistic competition between cells, which can be affected by conditions such as medium flow rate and initial bacterial inoculum.^67^ This antagonistic effect between eukaryotic cells and bacteria could be the key to a successful strategy relying on the use of antimicrobial nanocoatings; preventing bacteria from initially establishing dominance on surfaces while allowing cells (*e.g.*, host cells in the case of implanted biomaterials) to adhere.

The use of implanted biomaterials and medical devices continues to increase year upon year mainly because of the aging population and advancement of medical engineering. Implants may include joint replacements, internal fixation orthopedic implants (*e.g.,* screws, pins, plates), bone cements, dental and maxillofacial implants, tissue engineering scaffolds, artificial heart valves, pacemakers, stents, catheters, and wound dressings. Despite their high success rate, implants can still fail because of lack of biocompatibility and immunological rejection. However, development of peri-implantitis, which is caused by infection, remains the most common reason for implant failure.^68^ Infections caused by colonization of medical or dental implants can result in patient morbidity and mortality, as well as the need for repeated surgeries with associated financial cost, patient distress and wasted resources. Application of suitable nanocoatings to the surface of implants could offer IPC through inhibition of bacterial colonization and biofilm formation.^69^ Around half of all nosocomial infections are associated with indwelling medical devices,^70^ and in addition to the medical devices that come in direct contact with human tissues, there are a wealth of other surfaces in a clinical environment which can serve as reservoirs of pathogenic microbes. Examples include high-touch surfaces such as preparation surfaces in hospital kitchens and operating theaters, door handles, bedrails, taps, bedding, patient gowns, and scrubs. Detergents and disinfectants are currently used to improve hospital cleanliness but clearly have failed to eliminate the problem,^71−73^ and resistance is emerging.^74,75^ Applying durable antimicrobial nanocoatings to those surfaces could reduce the spread of infection since they could offer a long-lasting effect.

**Figure 3:**
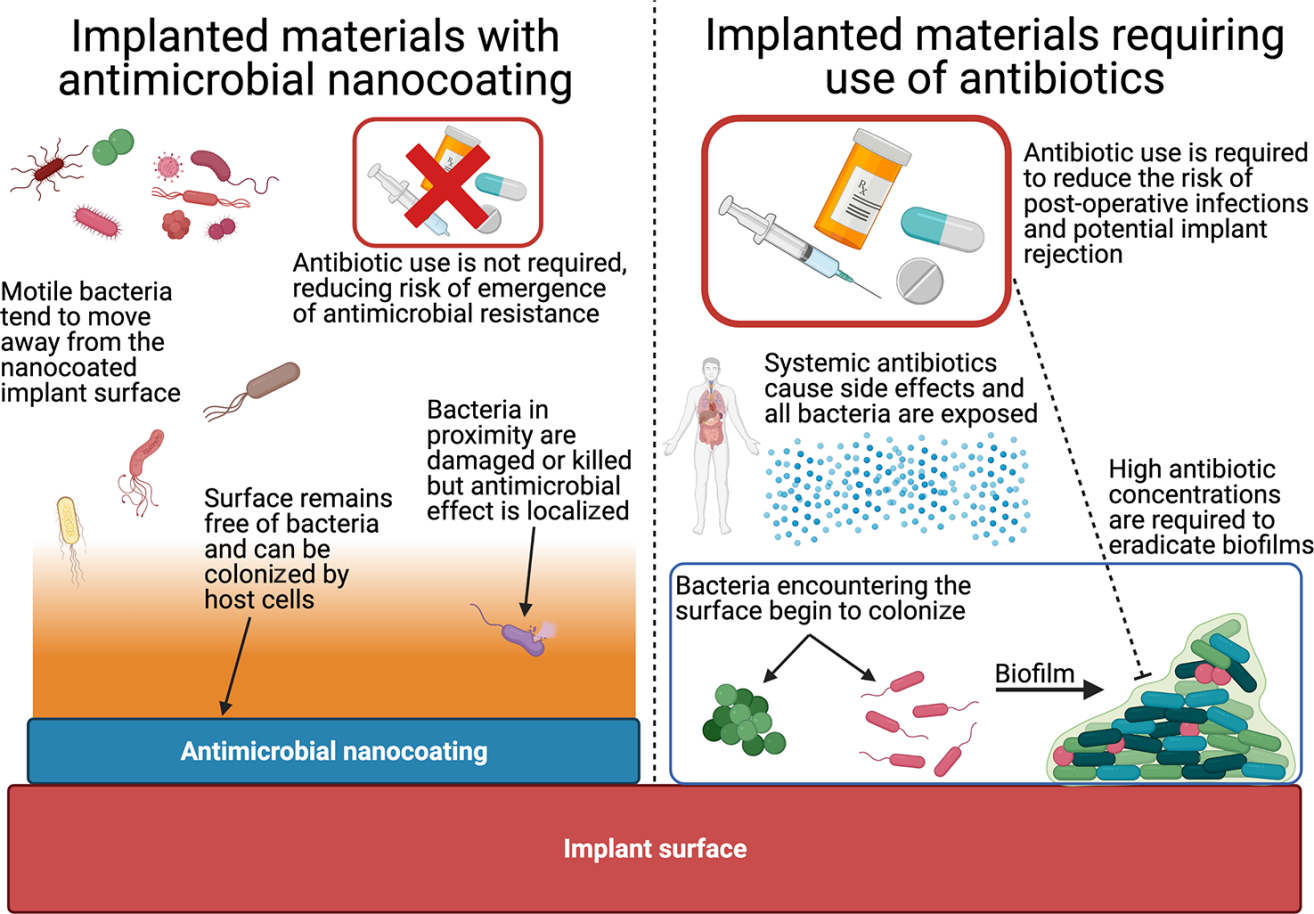
Advantages of implanted materials incorporating antimicrobial nanocoatings over those without. Implanted materials without antimicrobial nanocoatings become colonized by bacteria encountering the surface and forming biofilms. Prophylactic antibiotics are often given systemically, bringing side effects to the patient and exposing all bacteria to antibiotics, raising the risk of antimicrobial resistance. High doses of antibiotics are also required to eradicate mature biofilms. When antimicrobial nanocoatings are incorporated on the surfaces of implanted materials, the surface remains uncolonized and antibiotic use may not be required, leading to fewer patient side effects and less exposure of bacteria to antibiotics. While the antimicrobial effects of nanocoatings are local compared to systemic antibiotics, nanocoatings exert an antimicrobial effect beyond the immediate surface and cause bacteria nearby to move away or become damaged. Figure was created using BioRender.com.

In the context of implanted biomaterials, antimicrobial nanocoatings offer advantages over antibiotics (Figure 3). One advantage is the exertion of effects locally rather than systemically, as the immediate surface is protected by the nanocoating while other tissues distant from the implant site are not exposed to the antimicrobial. Related to this, nanocoatings may improve the patient experience by avoiding the side effects^84^ and complications^85^ of antibiotics. Furthermore, antimicrobial nanocoatings would facilitate a reduction in antibiotic usage, allowing them to be reserved for other essential therapeutic applications, and may thereby reduce the opportunity for selection of antibiotic resistant bacteria.

## Factors Affecting Antimicrobial Activity of ENMs

The activity of nanoparticulate metals differs from that of their bulk counterparts with factors such as NP size, shape, surface charge and elemental composition, playing a pivotal role in not only their physical and chemical characteristics but also their antimicrobial behavior. Due to the differing dimensions among published research and frequent lack of a systematic or easily comparable approach, it can be difficult to determine which properties confer the most potent antimicrobial effects. In some cases, it is difficult to conclude which properties are optimal given that different authors tend not to compare the same conditions, and thus there is generally a lack of direct replication of studies. It is clear though that there are complex interactions between size, shape, method of production and exposure conditions which affect overall antimicrobial activities. A greater understanding and appreciation of these properties and their combined effects on ENM antimicrobial activity will allow better fine-tuning of effects and improve suitability of ENMs to their applications.^86^

### Size

Baker et al.^87^ investigated the effect of size on antibacterial activity in silver NPs and found that NPs with a mean size of 15 nm exhibited higher antibacterial activity against *Escherichia coli* compared to those of 75 nm. Bactericidal properties against other Gram-negative bacteria including *Pseudomonas aeruginosa*, *Vibrio cholerae*, and *Salmonella* Typhi have also been found to be optimal for particles having a diameter of approximately 1–10 nm.^88^ The trend of antimicrobial activity increasing with decreasing NP size has been confirmed by multiple other studies.^89−91^

### Shape (Particle Morphology)

In general, spherical NPs are the most common, but other shapes including rods, cubes, flakes, and tubes are available. Some authors suggest that triangular nanoplates have the strongest biocidal activity,^92^ while others suggest cubic NPs are the most effective due to the exposed planes.^93^ The differences in activity related to particle shape or morphology appear to be due to variations in ionic release as an expression of the total surface area.^94^ Thus, particle morphology could be a valuable variable used to tune nanoparticle effects for intended applications, facilitating a controlled design.^86^

### Surface Charge

Zinc oxide NPs with a positive surface charge have been shown to exhibit antimicrobial activity against both Gram positive and Gram-negative bacteria, while NPs of the same size but with negative surface charge did not exhibit any inhibition of bacterial growth. This is hypothesized to be due to the positive surface charge of the NPs enhancing ROS production and applying mechanical stress on the negatively charged bacterial membrane.^95^ Zwitterion-modified silver NPs have been shown to be designed to shift their surface charge in response to differing pH conditions, allowing more targeted antimicrobial activity. This is achieved by NPs responding to physiological pH in healthy tissues while adhering to negatively charged bacteria at infectious sites with lower pH values.^96^

## Types of Nanocoatings

**Figure 4:**
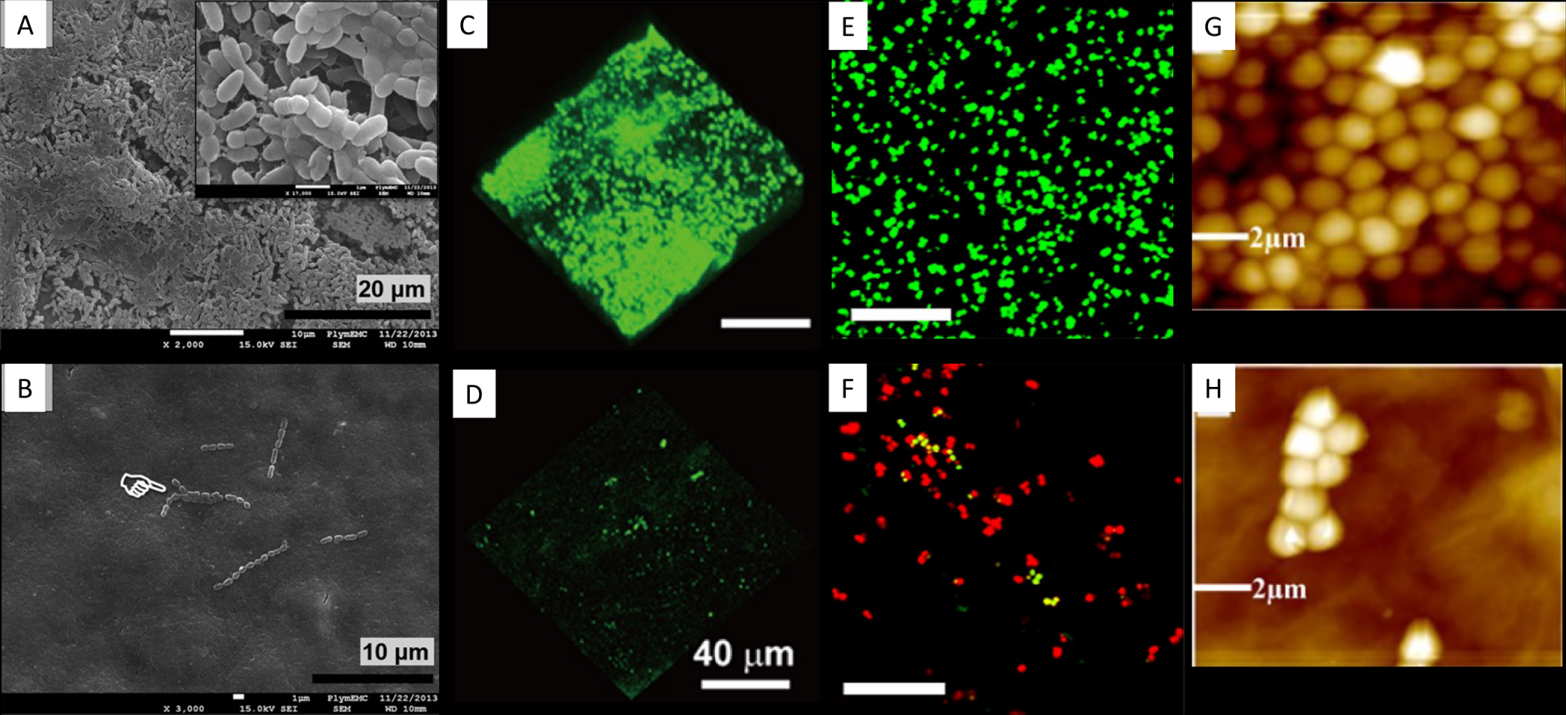
Representative examples of nanocoated surfaces showing antimicrobial activity compared to uncoated controls. (A) *Streptococcus sanguinis* biofilm on the surface of uncoated control titanium alloy implants compared to (B) absence of biofilm formation on those implants following application of a dual layer silver-hydroxyapatite nanocoating. Reprinted with permission from ref (69). Copyright 2017 Taylor & Francis Ltd. (C) Confocal laser scanning microscopy live/dead image of *Streptococcus mutans* biofilm on uncoated Invisalign aligners compared to (D) reduced biofilm formation on gold nanocluster-coated aligners. Reprinted with permission from ref (129). Copyright 2020 American Chemical Society. (E) Confocal laser scanning microscopy live/dead image of *Staphylococcus aureus* biofilm on control surfaces compared to (F) surfaces coated with stearic acid nanostructures where fewer live bacteria and far more dead bacteria were present. Reprinted with permission from ref (130). Copyright 2017 Elsevier. (G) Atomic force microscopy image of *Staphylococcus aureus* biofilm on a control surface compared to (H) limited biofilm formation on surfaces coated with graphene oxide. Reprinted with permission from ref (131). Copyright 2017 American Chemical Society.

Nanomaterials can vary significantly in shape, size, elemental composition, synthesis, presentation, and surface modifications, which means that there is a wide range of different types of nanocoatings available or currently under development. In this review, nanocoatings have been classified according to the family of materials they consist of: metal and metal oxide NPs (Table 1), ceramic, and nonmetallic NPs, including carbon-based nanomaterials (Table 2). Representative example images from studies describing antimicrobial nanocoatings can be seen in Figure 4.

**Table 2:** Summary of Additional Selected Examples from the Published Literature Regarding Application of Nonmetallic Nanomaterials as Antimicrobial Nanocoatings

Nanocoating description	Aim of application	Key methods^a^	Target organisms	Key results^a^	Source
Dual layer cotton coated with chitosan nanofibers incorporating *Agrimonia eupatoria* extract	Therapeutic wound dressings	Agar slurries of bacteria spread on surfaces	*Pseudomonas aeruginosa, Staphylococcus aureus*	Coatings with and without extract inhibited bacterial growth	(214)
		Slurries released and bacteria enumerated		Inhibition was greater (>98%) in extract-containing group	
		MTT used for *in vitro* cytotoxicity against human dermal fibroblasts		No cytotoxicity seen against human dermal fibroblasts	
Graphene nanoplatelets incorporated in polyurethane by melt blending and dip coating	Catheters	Bacterial culture deposited on surfaces	*Staphylococcus epidermidis*	Dip-coated surfaces caused 50–100% decrease in metabolic activity and 57–82% decrease in viability	(215)
		Nonadherent bacteria enumerated after 24 h		Bactericidal activity upon contact not shown toward adherent or planktonic cells for melt blended samples	
		Adherent bacteria visualized by FM			
Composite of graphite nanoplatelets and low-density polyethylene produced with graphite nanoplatelets oriented in a controlled manner, exposed by etching	Antibacterial surfaces for biomedical devices	Surfaces seeded with bacteria	*Escherichia coli, Staphylococcus epidermidis*	Bactericidal activity against *S. epidermidis* (95%) and *E. coli* (90%) was dependent on nanoflake orientation and density - in one group viability loss was >99.99% for both bacteria	(216)
		Bacteria enumerated after 24 h		Reduced bacterial adhesion and surface colonization seen with live/dead staining	
		Surfaces investigated with SEM, live/dead and reactive oxygen staining and FM		No toxicity against SH-SY5Y or HUH-7 cells	
		*In vitro* cytotoxicity evaluated against SH-SY5Y and HUH-7 cells			
Graphene coating developed using a one-step laser-induced method	Antibacterial coatings for water robots	Samples placed on agar plates inoculated with bacteria followed by 10 min solar treatment	*Escherichia coli*	>99.8% reduction in viable bacteria	(217)
		Bacteria enumerated after 12 h		SEM showed no bacteria on graphene-coated glass samples but dense ones on untreated surfaces	
		SEM to visualize samples			
Hydroxyapatite coating on titanium by microarc oxidation followed by loading of chitosan by dip-coating	Titanium implants	Surfaces seeded with bacteria and enumerated after 24 h	*Escherichia coli*	Up to 65% antibacterial activity demonstrated and improved with increasing chitosan but decreased over time	(218)
		Agar diffusion and UV spectrophotometry		No cytotoxicity or morphology differences found	
		Biocompatibility and osteogenic properties evaluated with MC3T3-E1 cells		Cells proliferated faster on surfaces lacking chitosan and cell numbers decreased as chitosan content increased	
Titanium implants coated with chitosan and alginate by spin coating	Titanium implants	Bacterial viability quantified following surface contact	*Escherichia coli*	Antibacterial activity of >36% demonstrated and improved with chitosan and alginate	(219)
		*In vitro* cytotoxicity against L929 fibroblasts		No cytotoxicity and biocompatibility demonstrated	
Graphene oxide (GO) applied to polyvinylidene fluoride by vacuum filtration	Antibiofouling coatings in wastewater treatment technologies	Bacterial cultures filtered through GO-modified membranes	*Escherichia coli, Staphylococcus aureus*	>70% bactericidal activity against both target bacteria with effects increasing over time	(220)
		Cell morphology observed by SEM		Bactericidal activity *S. aureus* ≫ *E. coli*	
		Surfaces seeded with bacterial suspensions for biofilm assays		SEM showed bacteria on coated membranes had compromised cellular integrity	
		CVSA quantification after 24 h		Reduced biofilm biomass on coated versus uncoated membranes	
GO applied to polyetheretherketone by dipping method	Bioactive implant material for bone tissue engineering	Immersion in bacterial culture and adhered cells enumerated after 24 h	*Escherichia coli, Staphylococcus aureus*	Changes in morphology and suppressed colonization for *E. coli* by 54–77% but not for *S. aureus*	(221)
		Fixed samples viewed with SEM		GO coating may enhance cytocompatibility as osteogenesis markers upregulated	
		CCK-8 kit and CLSM for *in vitro* cytotoxicity toward human osteoblast-like MG-63 cells			
Polycarbonate urethane was coated with a thin film of GO	Medical implants	Immersion in bacterial culture and adhered cells enumerated after 24 h	*Pseudomonas aeruginosa, Staphylococcus aureus*	No GO leaching detected	(222)
		GO leaching and hemolysis (ISO 10993–4) measured		Reduced adhesion of *S. aureus* (86%) and *P. aeruginosa* (64%)	
		*In vitro* adhesion and proliferation measured with L929 fibroblasts by FM and MTT assay		GO coating induced hemolysis more than controls (0.055% vs 0.02%), but was <5% (ISO 10993–4)	
				L929 cell proliferation unaffected compared to control	
GO-coated surfaces were prepared by Hummers’ method and an “improved” method	Antimicrobial surface coatings for biomedical applications	Immersion in bacterial culture for 24 h with viable cell enumeration	*Escherichia coli, Staphylococcus aureus*	Antibacterial activity seen in all treatment groups	(223)
		Biofilm assays in GO plated 96 well plates CVSA and microscopic visualization and quantification after 24 h		Smoother surface in “improved” method–fewer pores and less *E. coli* biofilm inhibition	
				Increasing GO content increased *E. coli* biofilm inhibition by up to 150% but not *S. aureus*, remaining around 75%	

a CVSA: crystal violet solubilization assay; CLSM: confocal laser scanning microscopy; SEM: scanning electron microscopy; FM: fluorescence microscopy; UV: ultraviolet; MTT: 3-(4,5-dimethylthiazol-2-yl)-2,5-diphenyltetrazolium bromide; CCK: cell counting kit.

### Metal and Metal Oxide Nanocoatings

Metal compounds have been used as antimicrobial agents since antiquity with silver, zinc, titanium, copper and gold having received the most interest, each showing different properties and antimicrobial efficacy.^97^

#### Silver Nanocoatings

Bulk metallic silver (Ag) has been known for its inherent antimicrobial properties since 4000 BCE,^98,99^ well before the introduction of the first antibiotics. More recently, Ag has been used in medical devices such as wound dressings and catheters to restrict or impede bacterial growth and biofilm formation.^100−102^

Silver nanoparticles (Ag NPs) applied as nanocoatings have been investigated in the context of medical implants and prostheses. In the oral cavity, bacteria must adhere to surfaces and form biofilms to survive and proliferate; nutrients in aqueous environments tend to accumulate on surfaces, and adhesion allows bacteria to resist the shear forces of salivary fluid movement and passage to the gastrointestinal tract beyond.^103^ As such, prevention of initial bacterial adherence and biofilm formation or reducing the rate of biofilm development and maturation appear to be the major goals of antimicrobial surface coatings in dentistry. Ag NPs have been applied in the form of nanocoatings directly to the surface of dentine.^77^ The Ag nanocoatings were found to be stable in biological fluids, prevent biofilm formation, and inhibit bacterial growth in the surrounding media. Ag NPs in this form were also found to be more bactericidal toward the oral pathogen *Streptococcus mutans* when compared to the oral disinfectant chlorhexidine. Despite Ag NPs being equally as bactericidal as silver nitrate (AgNO_3_), they did not cause dentine discoloration. Similar nanocoatings were later studied following application to titanium alloy orthopedic medical implants; silver-plated discs exhibited the highest antibacterial activity and strongest antibiofilm activity while experiencing very little material loss as a result of silver dissolution from the nanocoatings.^69^ Ag nanocoatings applied to the surface of silicone maxillofacial prostheses were found to prevent fungal infection caused by *Candida albicans in vitro,* while being highly biocompatible with dermal fibroblasts.^104^ These studies have demonstrated that application of silver nanocoatings to medical implants and tissues is a promising alternative antimicrobial strategy that also addresses potential biocompatibility issues. However, it should be noted that these studies were exclusively performed *in vitro*.

Meran et al.^104^ suggested good compatibility between Ag NPs and eukaryotic cells, a critical issue in nanomaterial development for clinical applications. A major advantage of Ag NPs is their low toxicity to mammalian cells relative to their bactericidal concentration. This means that although it is possible for them to be toxic to mammalian cells, this can only be possible at concentrations higher than those required to demonstrate bactericidal activity. The minimum inhibitory concentration (MIC) or minimum bactericidal concentration (MBC) of Ag NPs can be difficult to reliably determine using visual methods alone and turbidity corrections must be made because turbidity caused by NP dispersions can mask absorbance caused by bacterial growth at NP concentrations above 12.5 μg mL^–1^.^62^ Corrections are particle-specific as NP properties such as size, shape and crystallinity affect measurements of absorbance. Reported MIC values for Ag NPs include 67 μg mL^–1^ against a 6 × 10^5^ CFU mL^–1^ inoculum of *Streptococcus mutans*(105) or 4.9 ± 2.7 μg mL^–1^ against a 1.5 × 10^5^ CFU mL^–1^ inoculum of the same bacterium and an MBC of 6.25 μg mL^–1^.^106^ These findings highlight the extent to which seemingly similar studies can provide quite varying results, with proteins present in different growth media and the resulting protein corona effect being responsible for those differences.^107,108^ An MBC of 6.25 μg mL^–1^ has also been reported for the pathogens *Listeria monocytogenes*, *Escherichia coli* O157:H7, *Salmonella* Typhimurium, and *Vibrio parahemolyticus*.^109^ The minimum concentration having damaging effects on eukaryotic cells has consistently been found to be above 5 μg mL^–1^ in different cell lines.^110−112^ Other studies on Ag NPs have reported much higher values as minimum cytotoxic concentrations: 30 μg mL^–1^^113^ or even 61 μg mL^–1^.^114^ These data suggest that there is likely to be a sufficient window of concentration within which to design nanocoatings with appropriate nanoparticle release profiles. A balance must be achieved, generating a high enough concentration of Ag NPs in the local environment to have sufficient effect on bacteria without causing such a high material release as to lead to host cell toxicity or local tissue damage.

The balance of robust antibacterial efficacy with minimal toxicity to eukaryotic cells has been investigated using a porous poly(methyl methacrylate) (PMMA) substrate, a biomaterial highly susceptible to bacterial colonization, combined with a coating of immobilized Ag NPs.^76^ The Ag NP thin film was applied using pulsed laser deposition, a process optimized by varying the total laser pulses to alter the thickness of the film. The study showed that it was feasible to develop a manufacturing process to apply the optimal amount of Ag NPs to a PMMA medical implant, minimizing the risk of bacterial colonization while simultaneously reducing the risk of a patient adverse reaction.

Agnihotri et al.^115^ investigated Ag NPs immobilized on a functionalized silica surface. Their findings underscored contact killing as the predominant bactericidal mechanism in this context, and showed that immobilized (*i.e.*, surface-coated) NPs demonstrated greater efficacy than colloidal NPs of the same size and morphology. The tested Ag NP-glass surface was shown to be bactericidal for all three bacterial strains (two of *Escherichia coli* and one of *Bacillus subtilis*) investigated at both initial bacterial densities (10^3^ and 10^5^ CFU mL^–1^), and complete disinfection (quantified by viable counts of zero in duplicate) was achieved within 2 h for all test conditions. As would be expected, a higher initial bacterial load resulted in a longer time to disinfection, highlighting the importance of standardizing and reporting this value in future work. It was also found that coated surfaces could be reused many times without loss of antibacterial activity; complete disinfection of an initial bacterial load of 10^3^ CFU mL^–1^ of *Escherichia coli* was achieved within 50 min, even when the surface was used for the 11^th^ time. Disinfection was still achieved even when ionic silver release from the surface was very low (0.0109 μg mL^–1^).^115^ It would be valuable to investigate how more relevant physiological media containing proteins and other solutes can affect the efficacy of this type of coating; it is well-known that exposure of Ag NPs to physiological media containing proteins and other biomolecules compared to deionized water tends to cause greater agglomeration and more pronounced loss of antimicrobial activity,^116^ though this effect may potentially be circumvented by surface coating treatments aiming to prevent NP agglomeration. The relevance of this effect also depends upon the intended application of the nanocoating; it would be less relevant in a setting where nanocoatings are applied to clinical environmental surfaces (*e.g.*, bed rails, hospital fabrics, and door handles) compared to implanted medical devices that are exposed to high concentrations of biomolecules.

#### Copper Nanocoatings

Similar to Ag, copper (Cu) has been known to have biocidal effects since antiquity, with some more recent applications testing its use for high-touch surfaces (*e.g.*, door handles, bathroom fixtures, and hospital bed rails)^117^ and fabrics.^118^ Despite the encouraging evidence on antimicrobial activity, concerns about the toxicity and ecological impact of Cu NPs are likely to be the reason preventing further investigation of their use as antimicrobial nanocoatings. There is evidence that Cu NPs are toxic to mammalian somatosensory neurons, with greatest toxicity resulting from smaller NP size and higher concentrations.^119^ This is a significant finding as nanomaterials can be transported in a retrograde manner from nerve endings in skin to neurons in the dorsal root ganglion.^120^ Additionally, Cu NPs have been reported to be acutely toxic to zebrafish (*Danio rerio*), with the gill being the primary target,^121^ and cause retardation of zebrafish embryonic development and morphological malformation of larvae.^122^ The fate of Cu NPs in the environment is also different to that of other nanomaterials. For instance, sulfidation of CuO NPs to form Cu_2_S or CuS in environments with augmented sulfide levels, such as in wastewater treatment plants, increases solubility rather than decreasing it (as is the case with Ag and ZnO NPs) and leads to greater Cu^2+^ release,^123^ therefore resulting in increased toxicity to aquatic organisms.^124^

Despite the ecotoxicity concerns around Cu nanomaterials, there have been reports on their use as part of antimicrobial strategies. In order to combine and maximize the effects of the high surface area to volume ratio of NPs and the high aspect ratio of MWCNTs, CuO NPs have been investigated as an application to MWCNTs in contact with eukaryotic cells. Additionally, decoration of CuO NPs onto MWCNTs was anticipated to limit absorption of NPs by the human body as well as reduce the loss of NPs to the environment, addressing the ecological concerns to some extent. Mean average sizes of both cupric (CuO) and cuprous (Cu_2_O) NPs were <10 nm, which is generally at the smaller end of the spectrum for NPs. Cytotoxicity to human dermal fibroblasts only occurred at a relatively high concentration of 150 μg mL^–1^, while at 100 μg mL^–1^, some changes to cell morphology were observed but the proportions of live and dead cells remained unaffected. Comparatively, marked antibacterial activity was observed at 60 and 50 μg mL^–1^. Biofilm development by *Methylobacterium* spp. was inhibited at biocompatible concentrations, and furthermore the CuO/MWCNTs effectively managed to remove preformed biofilms. This study invokes optimism for the synergistic bactericidal and antibiofilm properties of CuO NPs decorated on MWCNTs.

Other studies have confirmed the potent antibacterial activity of CuO NPs when applied as coatings against *Escherichia coli*,^125^*Staphylococcus aureus*,^126^ and *Pseudomonas aeruginosa*(127) as well as antibiotic resistant bacteria such as methicillin-resistant *Staphylococcus aureus*.^128^ LewisOscar et al.^127^ demonstrated that CuO NPs had a strong antibiofilm effect, with a maximum of 94% biofilm inhibition against clinical strains of *Pseudomonas aeruginosa*, at a concentration of only 0.1 μg mL^–1^. This relatively low concentration demonstrating such potent antibiofilm effects makes CuO NPs an attractive option for antimicrobial nanocoating development. In addition, CuO NPs at that concentration inhibited the production of EPS by up to 93%, complementing the principal antibiofilm properties by preventing the formation of a protective EPS layer by the proportion of bacteria able to form a biofilm. These findings highlight the potent antibiofilm properties that CuO NPs have and which would be highly relevant to antimicrobial nanocoatings.

#### Gold Nanocoatings

Gold (Au) has been used as an anti-inflammatory agent for the chronic inflammatory disease rheumatoid arthritis, specifically as a disease-modifying antirheumatic drug. However, use of Au salts was replaced by alternative drugs in the 1990s due to adverse effects, limited efficacy and slow relief of symptoms.^132^ While this was a setback for the use of Au in modern medicine, more recently Au has been reconsidered for use in nanomedicine.^133^ Research suggests Au NPs have potentially reduced relative toxicity and lower masses required to achieve therapeutic efficacy, which makes them an attractive option. However, other studies have exhibited opposing results disputing their potential for clinical use.

Au NPs have been reported to lack inherent antibacterial properties altogether^134,135^ or to inhibit biofilm formation without having toxic effects against pathogens.^136^ Other studies have suggested that they do have an antibacterial effect, but this is weak, with high MIC values measured (*e.g.*, 197 μg mL^–1^ against *Streptococcus mutans*).^106^ Another study found no concentration-dependent effects of Au NPs against *Escherichia coli*, but did report that Au NPs affected cell division.^137^ There is evidence that Au NPs have antifungal effects, with one study reporting excellent size-dependent antifungal activity against *Candida* isolates.^138^

Presumably due to their lack of clear potent antibacterial effects, there is little evidence in the published literature describing the use of Au NPs in antimicrobial coatings. Adsorption of Au NPs on a silica surface tested against *Escherichia coli* and *Staphylococcus aureus* did not demonstrate any bactericidal properties.^139^ Au NPs can be applied as a shell around a dielectric core to produce an Au nanoshell, while these structures are physiologically inert, they can have photothermal effects and generate significant heat by their strong surface plasmon resonance. The plasmon resonance can be tuned to different wavelengths by varying the relative size of the dielectric core and the thickness of the Au layer.^140^ These Au nanoshells were applied to a silicone catheter surface and tested for antimicrobial activity against a drug-resistant strain of *Enterococcus faecalis* using a near-infrared diode laser to produce heat with potentially bactericidal effects. Application of the laser for 5 and 10 min resulted in severely diminished surviving bacterial numbers, with scanning electron microscopy showing thermally induced rupturing of bacterial cell walls.^141^ The success of the nanoshell coating becoming antimicrobial upon exposure to the near-infrared laser suggests a possible mechanism where segments of silicone catheter or other materials could be coated and subsequently sterilized on a regular basis. The comparative effects on bacteria in biofilms should also be investigated, though due to the physical method of bacterial killing, it is unlikely that biofilm formation alone would protect bacteria from the relatively high local temperatures (73 °C) encountered.

There is another field of research examining the use of Au NPs in combination with other molecules to deliver an antimicrobial effect, for example, by doping Au NPs with a tRNA analogue,^142^ loading them with 5-fluorouracil, an anticancer drug,^143^ or by coating them with the antibiotic amoxicillin.^144^ This type of application has previously been reviewed^145^ and is beyond the scope of this review because in those cases, the nanomaterial itself was not the active antimicrobial but acted as a carrier for drug delivery.

#### Zinc Nanocoatings

Zinc NPs are most used as antimicrobials in the form of zinc oxide (ZnO). A proposed benefit for ZnO nanocoatings applied to orthopedic or dental implants is the effect of zinc in augmenting bone formation by stimulation of osteoblast activity and cell proliferation.^146,147^ Zinc also has a role as a cofactor for collagen synthesis, and supports bone mineralization via alkaline phosphatase.^148^ This strong association with bone formation and mineralization makes ZnO NPs ideal candidates for use in antimicrobial nanocoatings near calcified tissues, such as bone scaffolds and joint replacement implants. The antimicrobial effects of ZnO NPs appear to be high, albeit potentially dependent on the morphology of the nanocoating. The strongest antimicrobial effect has been observed for nanomaterials with rod-like morphology and a high degree of crystallinity.^149^ These findings were contradicted by another study^150^ showing that ZnO nanocoatings had strong antibacterial activity toward *Escherichia coli* and *Staphylococcus aureus*, but no significant differences between particle morphologies were observed. Light-producing biosensor versions of the bacterial cells acting as reporters (constitutively expressing the *Lux* operon and emitting a light signal correlating with cell numbers) allowed real-time measurement of the antibacterial effect, demonstrating that a long incubation was not necessary; the antibacterial effects of ZnO nanocoatings were apparent even after short exposure times. Antibacterial effect also increases with thicker films of NPs, affecting the bacterial generation time and essentially retarding growth and leading to fewer bacterial cells present.^151^ Thicker films consist of larger quantities of NPs and presumably result in higher local concentrations of ions following NP dissolution.

Despite ZnO NPs showing good bactericidal efficacy, their biocompatibility and cytotoxic effects must also be considered. It has been reported that ZnO nanofilms significantly decrease cell viability (as confirmed by MTT assay) of cultured macrophages by 54% and 65% depending on NP size (100 and 20 nm, respectively) after a 48 h incubation, although no cytotoxicity was measured after 24 h.^152^ This initial lack of cytotoxic effect suggests a gradual release of material which accumulates over time to produce a more cytotoxic concentration, or alternatively could suggest a time-dependent cytotoxic effect. This contrasts with the alternative toxicokinetics where most material is released faster in the short term, causing higher toxicity in the early stages. A later report highlighted that direct exposure of cells to ZnO nanofilms could cause apoptosis and necrosis, two forms of both controlled and uncontrolled eukaryotic cell death, in a murine macrophage cell line.^153^ Depending on the type of bioassay employed (MTT versus LDH), cells grown on ZnO nanofilms showed a 43–68% loss of viability following a 24 h exposure compared to controls, with cells separately exposed to undiluted extracts from the coatings showing even greater viability loss. Two diluted coating extracts, 25% and 50% (corresponding to concentrations of 3.03 and 6.07 μg mL^–1^, respectively) showed no cytotoxic effects against macrophages, indicating a tolerable concentration of ZnO NPs, but it was unclear whether these concentrations would have an antimicrobial effect. Petrochenko et al.^153^ highlighted the importance of using both direct-exposure and extract-based methods to assess toxicity, as nanocoatings show gradual material release which can accumulate to a toxic level over time and extracts can simulate the result of this accumulation. It is difficult to draw wide conclusions based on these individual studies, but there are indications that ZnO nanocoatings could have inherent biocompatibility issues. Research efforts to address the biocompatibility of ZnO nanocoatings have been scant, with most studies looking at ZnO coatings at particle sizes greater than the nanoscale. A more recent study investigating the *in vitro* biocompatibility of ZnO nanofilms at the nanoscale found that direct exposure to ZnO nanofilms reduced cell viability of mouse fibroblasts due to inhibition of cell adhesion, regardless of ZnO crystallinity.^154^ This study appears to agree with that published by Petrochenko et al.^153^ and provides further evidence of the adverse effects of ZnO nanocoatings on eukaryotic (*i.e.*, host) cells, leading to concerns over the safety and biocompatibility of ZnO nanocoatings *in vivo*.

#### Titanium Nanocoatings

Titanium (Ti) and its alloys are the industry standard for implanted biomaterials due to their inherent biocompatibility, inert chemistry, strength, corrosion resistance, and lack of toxicity.^155^ Ti NPs have also been the subject of extensive research due to their well-established photocatalytic properties,^156^ further enhanced by the high surface area of NPs, providing antimicrobial properties.^157^ Titanium dioxide (TiO_2_) is most associated with this application, and exists in three main forms: anatase, rutile, and brookite. Anatase is the most photochemically active phase of TiO_2_, though combinations of different phases may show heightened activity compared to anatase alone.^158,159^ The high photoactivity and stability of TiO_2_, along with its relatively low cost and lack of toxicity has led to its consideration as a potentially self-disinfecting or self-sterilizing surface coating.^160,161^ An advantage of a photocatalytic self-disinfecting surface is that there is no necessity to add other chemical reagents; the only requirements would be oxygen, water and light.^162^ The band gap energy of anatase TiO_2_ is approximately 3.2 eV, corresponding to activation by photons with wavelength shorter than 385 nm and therefore to UVA light.^163^ However, since only 8% of solar radiation is UV, there is a need to develop photocatalysts which can be activated predominantly by visible light (42% of solar radiation), especially if a surface is intended for environmental use with activation by sunlight.^164^ The extent to which activation by sunlight is a relevant mechanism will depend on the intended application of TiO_2_ nanocoatings; activation of antimicrobial properties by the ambient lighting on a hospital ward or similar environment would be highly beneficial.

The nature of the antimicrobial mechanism based on ROS production suggests that TiO_2_ nanocoatings can be hostile to both bacterial cells and eukaryotes, limiting their use *in vivo*. Several studies have reported that TiO_2_ NPs exhibit toxicity, including evidence of genotoxicity, in both light and dark conditions.^165−168^ The production and toxicity of ROS are indiscriminate, and therefore there is a presumption that ROS are likely to damage all cells within the vicinity.^163^ Despite this, TiO_2_ nanocoatings have been investigated in a dental context, applied to orthodontic brackets.^169^ Brackets coated with nitrogen-doped TiO_2_ nanofilms were shown to cause significant CFU reductions over 90 days compared to uncoated brackets when tested with the oral pathogen *Streptococcus mutans*. To date, there is little robust evidence regarding safety of TiO_2_ nanocoatings to oral cells, but additional research exposing eukaryotic cells to these coatings and their associated ROS, over relevant time periods, will be crucial prior to clinical testing. However, it should also be remembered that TiO_2_ is already heavily used as an additive (E171) in the food industry^170^ as a mixture of micro- and nanosized particles for food coloring purposes. E171 has been found to induce ROS generation in a cell-free environment but not in exposed Caco-2 cells, induce single-strand DNA breaks and cause chromosome damage.^171^ However, no acceptable daily intake is currently defined in the European Union (EU) due to TiO_2_ bioavailability being found to be low and independent of particle size, the vast majority of TiO_2_ being eliminated unchanged in feces, and a maximum of 0.1% being absorbed by gut-associated lymphoid tissue and distributed to organs.^172^

Equally important to the development of implanted biomaterials utilizing a TiO_2_ photocatalytic surface is the longevity of antibacterial activity following cessation of UV irradiation. While environmental surfaces can be suitable for continuous or repeated photocatalytic activation where antibacterial effects are immediate but short-lived following cessation, this model may not be suitable for implanted biomaterials which are inaccessible. A nanocomposite of resin and TiO_2_ NPs demonstrated detectable antibacterial effects for 30 min following cessation of UV irradiation.^173^ The post-UV treatment effect was tested against five bacterial strains: *Escherichia coli*, *Staphylococcus epidermidis*, *Streptococcus pyogenes*, *Streptococcus mutans* and *Enterococcus faecalis*. Although UV treatment did not affect bacterial adhesion to coated specimens, the viability of bacteria was reduced by 37%. This finding is particularly relevant because the highest risk of bacterial colonization for implanted biomaterials is prior to or during implantation. Maintaining a UV-induced antibacterial effect for even 30 min following cessation of irradiation may allow enough time for implant surfaces to self-disinfect following implantation and reduce the possibility of biofilm development and subsequent infection, which can in certain cases result in implant failure.

#### Aluminum Nanocoatings

Aluminum oxide (Al_2_O_3_, also termed alumina) NPs have been shown to have some antimicrobial effects, albeit at very high concentrations (1000 μg mL^–1^) when tested against *Escherichia coli*.^174^ It was postulated that while they exhibit toxicity to bacteria through surface charge interactions with cell membranes and walls, their free radical scavenging properties may limit intense antimicrobial action and disruption of the cell wall. Essentially, they may simultaneously exhibit antimicrobial properties by one mechanism while reducing that antimicrobial effect by another. A similar MIC in the range of 1700—3400 μg mL^–1^ was reported for a multidrug-resistant strain of *Staphylococcus aureus*.^175^ More recent work has demonstrated antibacterial activity at a concentration 1 order of magnitude lower (100 μg mL^–1^) against both *Escherichia coli* and *Staphylococcus aureus*.^176^ The EC_50_ (half maximal effective concentration) of Al_2_O_3_ NPs against *Pseudomonas putida* has even been reported at 0.5 μg mL^–1^ over 16 h.^177^ The differences in values between these reports demonstrate the confounding factors of NP size, shape and synthesis method and suggest that they could be as important as concentration in terms of antimicrobial activity. Nevertheless, most studies investigate alumina NPs in the form of nanosolutions with very little evidence in the literature where they have been used as nanocoatings.

### Nonmetallic Nanomaterials

#### Carbon-Based Nanocoatings

There are a number of unique carbon-based nanomaterials (CBNMs), primarily allotropes of carbon such as graphene, with intrinsic antimicrobial properties and distinct material properties which make them useful for a range of applications in medicine and dentistry. A key property of CBNMs is their excellent biocompatibility, resulting in their testing in a range of biomedical applications including drug delivery, biosensor development, diagnostics and therapeutics.^186^ The various types of CBNMs available, in addition to graphene, include single- and multiwalled carbon nanotubes, fullerenes, and nanodiamonds.^187^

Graphene. Graphene consists of a single layer of carbon atoms arranged hexagonally and is the base component of materials including carbon nanotubes (CNTs), diamond, charcoal, graphite, and fullerenes (collectively referred to as graphene-based materials, GBMs). GBMs have intrinsic antimicrobial properties and appear to exert stronger effects if presented as coatings.^188^ Graphene can disrupt the bacterial cell membrane, most likely due to its physically sharp structure, interfering with the membrane potential and inducing membrane stress.^189,190^ While graphene in free-floating form exerts its bactericidal effect through both biomechanical interactions and ROS-mediated biochemical responses, surface-immobilization of graphene as a coating appears to limit the mechanism to primarily physical interactions causing cell membrane damage.^191^ Superoxide ion-induced ROS production does not appear to occur; however, oxidative stress can be produced by oxidation of glutathione, a redox mediator in bacteria.^192,193^ Like some other nanomaterials, the direct biomechanical mechanism of bactericidal activity offers the potential to be effective against drug-resistant pathogens, helping to protect surfaces from colonization. It has been suggested that graphene has antibacterial activity due to its ability to transfer electrons away from bacteria, as they maintain a negative resting membrane potential and require proper electron movement for the functioning of the respiratory chain.^194^ As graphene is an excellent electron acceptor, physical contact between bacteria and graphene may be sufficient to cause the bacteria to steadily lose electrons, interrupting the electron transport chain and leading to bacterial cell death. This effect also depends on the properties of the underlying substrate, in particular the substrate’s electrical conductivity.^195^ Research into the use of GBMs as antimicrobial coatings is still at a comparatively early stage, with relatively few publications available compared to the other groups of nanomaterials presented in this review; however, multiple methods of GBM application to relevant substrates have been reported.

Graphene was applied in the form of immobilized graphene nanoplatelets (*i.e.*, stacked graphene sheets with thickness of 2–10 layers) to the surface of silicone rubber to offer antimicrobial protection against *Staphylococcus epidermidis*. Independent of application methodology, the oxidized form of graphene had augmented bactericidal properties versus the nonoxidized form which may be explained by additional exertion of oxidative stress and production of ROS, leading to lipid peroxidation, mitochondrial dysfunction and protein inactivation.^196,197^ Graphene nanoplatelets have also been applied by spray coating onto a segment of silicone catheter.^198^ Spray coating has the advantage of simple adjustment of coating thickness by altering the number of passes of the nozzle over the sample surface. Dybowska et al.^198^ found that the graphene nanoplatelet coating was an effective antibiofilm agent preventing mature biofilm formation. However, graphene nanoplatelets decorated with Ag NPs were found to be even more effective indicating possible graphene-nanosilver synergism.

Other studies have investigated the potentially higher antimicrobial efficacy of graphene oxide (GO) nanocoatings. GO coatings have been applied to a polymeric substrate by immersion of plasma activated silicone films in a GO dispersion.^199^ Both colony counting and live/dead assay results showed considerable antibacterial activity against *Escherichia coli* and *Staphylococcus aureus*, with stronger activity against the former. That study concluded that the majority of bactericidal activity was the result of oxidative stress mechanisms, rather than physical or mechanical cell damage, due to the “edge-free” nature of the coating. However, this would not seem to eliminate possible antibacterial mechanisms involving interruption of electron transport. In a different study, GO-coated surfaces were prepared by two different methods, and effective inhibition of biofilm formation was reported for both *Escherichia coli* and *Staphylococcus aureus*.^131^ The synthesis method was a major factor affecting antibacterial efficacy, as different methods resulted in variations in functional groups present as well as nanosheet size, roughness, porosity, and thickness. These factors were significant as confirmed by the increased bacterial adhesion on the rougher nanocoating with less uniform thickness. In addition to GO coatings, reduced GO (rGO) coatings have been synthesized using the whole cell biomass of the fungus *Rhizopus oryzae*, coated on aluminum.^200^ Both the GO and rGO coatings showed excellent bactericidal activity against *Escherichia coli* (72% and 93% respectively), although their activity was lower than that shown for the same nanomaterials in a dispersed phase (80% and 97%); potentially because immobilization as a coating prevented access of the nanomaterials to intracellular compartments. Findings regarding bactericidal activity of the coatings were confirmed by live/dead assay, which also revealed reduced bacterial adherence to the rGO coatings and suggested that its more hydrophobic nature prevented cell attachment in addition to direct bactericidal activity. These findings indicate that GO and its variants have impressive potential to be used as antimicrobial nanocoatings, combining relatively facile and eco-friendly synthesis with potent antibacterial and biocompatible properties.

Carbon Nanotubes. CNTs are forms of graphene arranged in a cylindrical structure and can be structured with a single wall (SWCNTs) or multiple walls (MWCNTs). The single versus multiwalled nature is one of the variable properties of CNTs, along with diameter, length, surface functionalization (*e.g.*, addition of chemical groups), and chirality. There is a strong evidence base to support the antibacterial properties of CNTs,^201^ but only a few reports of applications as surface coatings. CNTs have been reported to be compatible with photodynamic antimicrobial chemotherapy, where light is used to activate or tune the antimicrobial effects. This approach has been shown to be effective against both *Staphylococcus aureus*(202) and *Escherichia coli*.^203^ Antimicrobial and antibiofilm activity have been suggested to be the result of ROS generation which allows antimicrobial photodynamic inactivation via cell membrane damage.

Carbon nanotubes have been applied as an antimicrobial coating to paper, which can widen the range of surfaces that can be coated to protect against bacterial colonization and transmission in healthcare settings.^204^ Direct interaction of bacteria with paper coated with acid functionalized SWCNTs for 1 h resulted in substantial morphological changes with loss of shape and integrity, explained by damaged cell walls leading to osmotic swelling. Both *Staphylococcus aureus* and *Escherichia coli* experienced these morphological changes, but those were more severe for *Staphylococcus aureus*; probably because of the greater rigidity of the *Escherichia coli* cell wall.

The mechanical properties of CNTs can also be useful in producing an antibacterial effect. Vertically aligned carbon nanotubes (VACNTs) have a very high aspect ratio with extreme flexibility, meaning that they deform in contact with bacteria before releasing their stored elastic energy. Arrays or “forests” of VACNTs with gaps smaller than the size of bacterial cells have been found to have potent bactericidal activity against *Pseudomonas aeruginosa* and *Staphylococcus aureus*.^82^ The proposed mechanism of action involves CNTs retracting and stretching in response to cell attachment, with release of the stored elastic energy resulting in tearing of the adsorbed bacterial cell. This mechanical killing mechanism is an attractive complement to other mechanisms involving oxidative stress or disruption of biomolecules, with the additional benefit of killing both Gram positive and Gram-negative bacteria.

#### Silica Nanocoatings

Silica nanoparticles (SiO_2_ NPs) have exhibited potent antibacterial effects expressed by high killing efficacy (>99%) against *Pseudomonas aeruginosa* and *Escherichia coli* biofilms, while demonstrating good clinical biocompatibility by inhibiting fibroblast proliferation less than conventional antiseptics.^205^ Attachment of SiO_2_ NPs to tissue culture polystyrene has been shown to reduce the attachment and growth of *Candida albicans*,^206^ and SiO_2_ NPs have also been found to be useful as abrasives for tooth polishing when tested on human teeth *ex vivo*.^207^

SiO_2_ NPs have been deposited as a coating on titanium substrates by an electrophoretic-enhanced microarc oxidation technique and tested against *Staphylococcus aureus* and *Escherichia coli*.^81^ The coated substrate showed slightly reduced bacterial growth, but cell morphology was the same when compared to uncoated substrates. Results showed that coated surfaces slightly inhibited bacterial adhesion and growth, but this effect was greatly enhanced by addition of octenidine, a cationic surfactant and antiseptic. The authors attributed the antibacterial properties of the SiO_2_ coating without octenidine to the highly porous structure of the surface, suggesting that bacteria became physically trapped which resulted in restricted movement and proliferation. This is analogous to the “trap-killing” previously reported against *Staphylococcus aureus* on Ag nanocoatings applied to titanium.^208^

Coatings of SiO_2_ NPs have been applied to tiles and tested for antifungal activity against *Acremonium kiliense*, *Acremonium strictum*, and *Fusarium solani*. Measurements of the fungal growth showed a reduction by 27.5%, 21.5% and 37.5%, respectively.^209^ Antifungal activity was also found to be higher for silica–titania core–shell NPs when compared to pure SiO_2_ NPs, suggesting that it was the layer of titania enhancing their antimicrobial performance.

#### Chitosan Nanocoatings

Chitosan is a polycationic polymer obtained commercially from shrimp and crab shell chitin by alkaline deacetylation, usually by sodium hydroxide.^210^ Both chitin and chitosan are biocompatible, biodegradable and nontoxic, though chitosan is favored due to its higher solubility and enhanced antimicrobial activity.^211^

A hybrid nanomaterial incorporating chitosan and silica was applied to the surface of titanium implants and tested as an antibacterial coating.^212^ Chitosan was the intended antibacterial component, whereas silica was selected for its osteogenic properties. The nanocoating was synthesized following the sol–gel process with chitosan covalently bonded to the silica network. Work using human fibroblasts demonstrated that the hybrid nanocoating was not cytotoxic, and cell proliferation was supported on the nanocoated surfaces, suggesting good biocompatibility. Significant antibacterial performance against *Staphylococcus aureus* was demonstrated for 5–10% chitosan, with antibacterial activity increasing with chitosan content. It is important to be aware of the hydrophilicity or hydrophobicity of any nanocoating, as this can impact directly upon interactions with the biological environment and dictate cell attachment.^213^ Palla-Rubio et al.^212^ found that adding chitosan decreased hydrophilicity of the coatings and reported contact angles for optimal biological interactions from 60 to 80°.

### Surface Nanotopography

Modification of surface nanotopography has been explored as an alternative antibiofilm strategy to the application of nanocoatings (Table 3). Certain nanotopography features, such as nanospikes or other controlled surface patterns, have been found to either hinder bacterial adherence or cause cell death by physically damaging the structure of the bacterial cell wall^224,225^ as well as by inhibiting cell division or by causing oxidative stress.^226^ The main advantage of this approach is that there are no biologically or chemically active substances involved that may leach from the surface over time, potentially causing local tissue or environmental toxicity and leading to long-term reduction or loss of antimicrobial efficacy. However, it is possible that nanopatterned surfaces may still become damaged following environmental exposure (*e.g.*, corrosion, abrasion), or nanotopographical details become masked by contaminants in the immediate environment. A natural buildup of biomolecules could potentially occlude the surface nanotopography and even enhance microbial adhesion; akin to the formation of a “conditioning film” which forms on abiotic surfaces upon contact with biological fluids containing proteins and polysaccharides and facilitates attachment of biofilm-forming cells.^227^ An additional caveat of modifying the surface nanotopography is the effect on biocompatibility. It is generally anticipated that rendering a surface inhospitable to bacteria by modifying its physical nanotopography would also affect the ability of eukaryotic cells to adhere and proliferate; a major issue for medical implants where the surface needs to be nontoxic while allowing integration with the adjacent host tissues. Some studies have reported that topographical features can affect immune cell function, raising questions about the indirect effect on biocompatibility and long-term host integration in addition to the more immediate effects on host cells. The morphology and spatial orientation of macrophages, key innate phagocytic cells with roles in determining downstream immune responses, are affected by topography and this may affect macrophage differentiation and the type and level of cytokine secretion. This suggests that physical cues, including surface topography, could modulate differentiation toward M1 (proinflammatory) or M2 (pro-healing/homeostatic) phenotypes.^228,229^ While this suggests that nanotopographical modifications should be applied with care, it could also present an opportunity for surfaces to be designed to stimulate a desired anti-inflammatory and pro-healing environment and thereby improve biomaterial integration.

**Table 3:** Summary of Additional Selected Examples from the Published Literature Regarding Surface Nanotopography Modifications Acting as Antimicrobial Nanocoatings

Nanocoating description	Aim of application	Key methods^a^	Target organisms	Key results	Source
Titanium/mica/glass surfaces modified using self-assembling nanostructures composed of fluorinated phenylalanine	Biofilm control for biomedicine	Immersion in bacterial suspension	*Enterococcus faecalis*	Viability (ATP production) reduced for *E. faecalis* (94%) and *S. mutans* (99%)	(252)
		ATP luminescence assay for viability after 24 h	*Streptococcus mutans*	Reduced bacterial adherence and metabolism affected	
		SEM to visualize biofilms			
A one-step etching technique was used to render aluminum alloys with micro- and nanoscale roughness	Engineered surfaces to minimize the spread of nosocomial pathogens	Immersion in bacterial suspension	*Escherichia coli*	97% lysis of adherent *E. coli* within 30 min	(253)
		After 4 h, nonadherent cells removed and samples incubated in broth	*Staphylococcus aureus*	28% kill of *S. aureus* attached to engineered surface versus 3% on control surfaces	
		SEM and live/dead staining with CLSM used to visualize bacteria after 20 h	“Nosocomial pathogens” recovered from patients	Disrupted morphology and >80% cell lysis for *P. aeruginosa* and *E. coli*, 25% for *K. pneumoniae*	
Metal organic framework nanodagger arrays	Safe and clean antimicrobial surfaces for medical devices	Surfaces seeded with microbial suspensions	*Escherichia coli*	Log reductions in viability for *E. coli* (7 log) *S. aureus* (8) and *Candida albicans* (4)	(254)
		Attached microbes enumerated after 18 h	*Staphylococcus aureus*	Good surface durability (for *E. coli*) over 2 months with no growth on treated surface vs 4 log on controls	
		Surfaces live/dead stained for visualization	*Candida albicans*		
		SEM to observe morphological changes			
Nanostructure arrays assembled from fatty acids assembled on graphite surfaces	Mechanobactericidal surfaces not requiring use of antibacterial chemicals	Immersion in bacterial suspension	*Pseudomonas aeruginosa*	>90% antibacterial activity against *P. aeruginosa*	(130)
		Aliquots taken for enumeration after 1, 3, and 6 h	*Staphylococcus aureus*	73→95% activity against *S. aureus*	
		Adherent cells visualized with live/dead stain and CLSM after 18 h		>90% of both bacterial species killed after 6 h in contact assays	
Diamond nanocone arrays fabricated by microwave plasma chemical vapor deposition then bias-assisted reactive ion etching	Biomaterials to reduce medical device-associated infections	Immersion in bacterial suspension	*Pseudomonas aeruginosa*	High proportions of cells with damaged membranes on nanopatterned surfaces	(255)
		Adherent cells visualized with live/dead stain after 1 h		Bacteria viewed in many orientations on nanopatterned surfaces–only horizontal on control surfaces	
		SEM for unstained surfaces			

a ATP: adenosine triphosphate; SEM: scanning electron microscopy; CLSM: confocal laser scanning microscopy.

It should be noted that some of the terminology in this area is not well-defined or standardized, with different publications describing types of nanostructures in different ways (*e.g.*, nanopillars, nanoneedles, nanospikes, nanocones). Due to this potential ambiguity, the more general term “nanoprotrusions” is used in this review.

#### The Role of Surface Roughness

There is general acceptance of the idea that bacteria are more likely to adhere to rougher surfaces due to the increased contact area, as well as the defects in the surface (pits, bumps or troughs) which provide protection from shear forces or contact abrasion with other surfaces (see review by Crawford et al.^230^). This has led to a common belief that smoother surfaces will reduce bacterial adhesion and so are the best strategy for inhibition of biofilm formation.^231^ However, a certain degree of nuance should be considered as there is some conflicting data from a range of studies which collectively find that surface roughness alone is not a good predictor of bacterial adhesion or colonization. Previous conclusions concerning surface roughness and bacterial adhesion have often failed to take into account bacterial appendages such as flagella which are able to aid in attachment by reaching crevices much smaller than the bacterial cells themselves^232^ (evidence reviewed by Mi et al.^233^). It could be that smoother surfaces are indeed more hostile surfaces for adhesion, but due to the very small size of these appendages, surfaces must be far smoother with fewer nanoscale defects than previously considered. Other factors such as surface free energy, wettability/hydrophobicity, surface chemistry and phenotypic differences between bacteria should also be considered central to affecting the likelihood of successful adhesion.^234^ There is also a dilemma for the development of surfaces intended for implantation *in vivo*; a roughness of 1–2 μm is deemed necessary for osseointegration and the long-term success of the implant.^235^ The relationship between surface characteristics or topography and biofilm development has been reviewed in detail by Teughels et al.^236^

#### Nanoprotrusions

**Figure 5:**
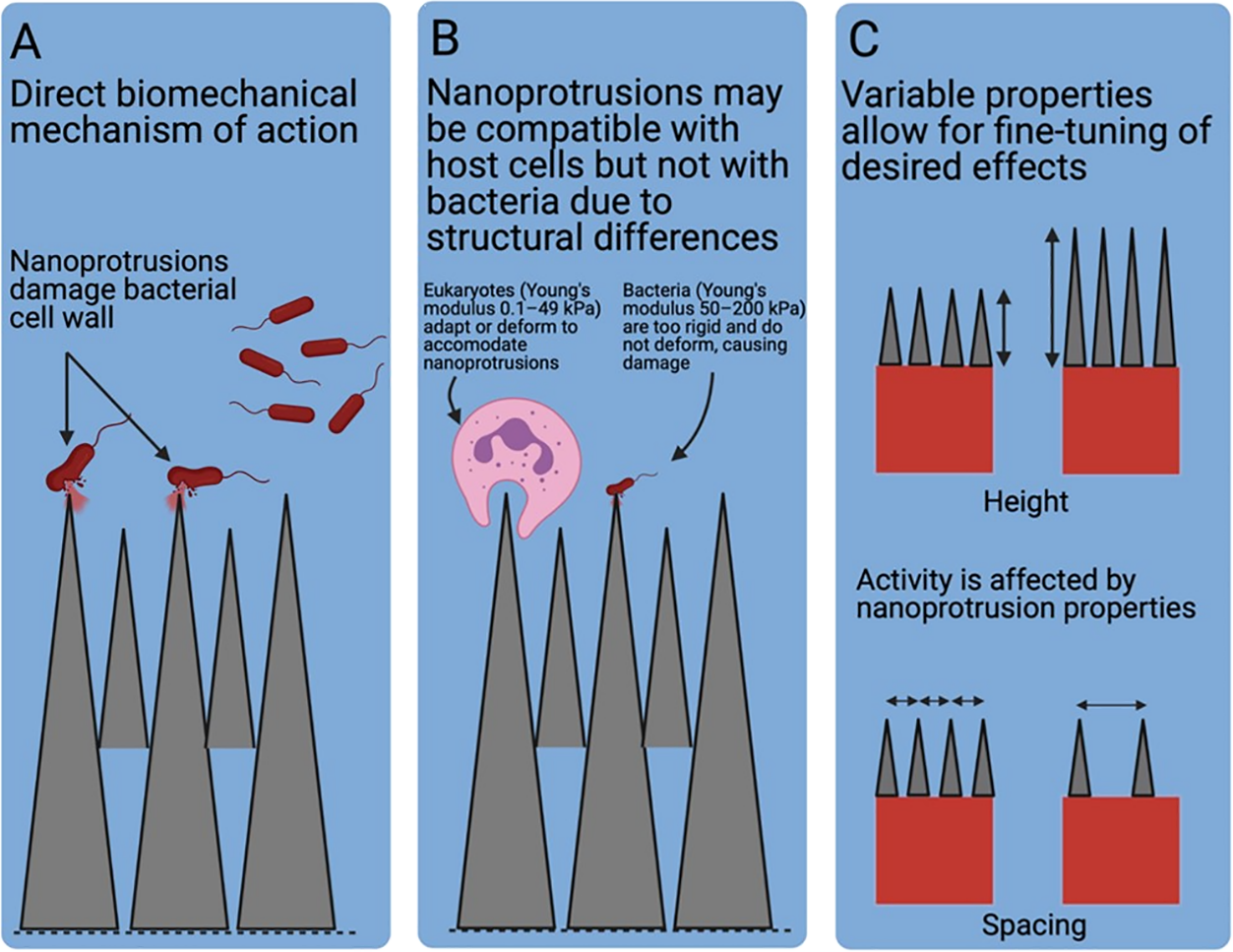
Advantages of nanoprotrusions and their potential to be fine-tuned. The direct biomechanical mechanism of action (A) of nanoprotrusions avoids possible concerns regarding resistance to antimicrobial agents, however the surfaces can be designed to be compatible with host cells while hostile to bacteria due to cellular structural differences as quantified by Young’s modulus (B). The antimicrobial and biocompatible properties of nanoprotrusions can be fine-tuned by modifying certain variables such as nanoprotrusion height and spacing (C). Features of graphic not to scale. Figure was created using BioRender.com.

Surfaces can be modified to have physical protrusions on the nanoscale similar to those observed in nature, for example on insect wings (cicada and dragonfly) and indeed natural nanotopography has been the inspiration for a number of biomimetic engineered surface modifications.^237^ These surfaces exert antimicrobial activity by direct biomechanical disruption of bacterial structures such as the cell wall or envelope^238^ by penetrating bacterial cell walls and causing irreversible cell damage (Figure 5A). Additionally, shear forces induced when bacteria move laterally relative to the nanoprotrusions increase the damage to the cell wall, also resulting in antimicrobial activity.^239^ The activity and specificity of nanoprotrusions may be dictated by their spacing and width (Figure 5C). In terms of bacterial variables, antimicrobial efficacy is thought to depend on cell shape and cell wall rigidity.^240^ Evidence suggests that the rigidity or stiffness of bacterial cell walls is significantly greater than that of eukaryotic cell membranes, with the Young’s modulus (a measure of resistance to elastic deformation where larger numbers indicate increased stiffness) of human mesenchymal stem cells in the region of 0.09–49 kPa compared to 50–200 kPa for certain bacterial cell envelopes, though it should be noted that these values are affected by cell type and viability or membrane integrity.^241,242^ This difference in cell wall rigidity between eukaryotes and prokaryotes could explain why some nanostructured surfaces facilitate eukaryotic cell proliferation but result in cell death for bacteria (Figure 5B). Eukaryotic cells have been shown to be able to stretch and distort to accommodate the shape of nanostructures, either growing around them or sitting on top and distorting to accommodate their shape, thereby avoiding membrane damage and cell death.^243^ When exposed to nanostructured surfaces, flexibility and adaptability appear to be superior to a rigid or stiff structure. Within prokaryotes, it has also been reported that Gram-negative bacteria are more susceptible to killing by nanopatterned structures such as cicada wings, with Gram positive bacteria showing greater resistance, presumably due to their thicker cell wall and differences in rigidity.^225^

Arrays of TiO_2_ nanowires have been shown to be selectively bactericidal against *Pseudomonas aeruginosa* with no activity against *Staphylococcus aureus*.^244^ Although this may be partly explained by the previous points regarding Gram-negative bacterial cell wall thickness, Diu et al.^244^ suggested that bacterial motility may also be associated with stronger bactericidal effects. Upon investigation with a panel of Gram positive and Gram-negative bacteria, significantly higher bactericidal activity was indeed found against motile versus nonmotile bacteria. Antimicrobial efficacy against *Staphylococcus aureus* has also been shown by using gold^245^ and titanium^246^ nanoprotrusions.

Black Silicon. Black silicon, a surface-modified variant of silicon produced by reactive-ion etching techniques, has been shown to kill a variety of bacteria (both Gram positive and Gram-negative) and endospores by surface contact.^225^ Nanoprotrusions of black silicon are sharper, more distinct, and approximately double the height of those found on a dragonfly wing. The high bactericidal efficiency of black silicon was particularly noteworthy, with a reported killing rate of ∼450,000 cells min^–1^ cm^–2^. Combining this evidence with known minimum infective doses (MIDs) for certain bacteria, one may conclude that 1 cm^2^ of black silicon could be capable of killing the MID of *Staphylococcus aureus* 810 times or that of *Pseudomonas aeruginosa* 77,400 times over 3 h. However, black silicon may not be as efficient against spores since it has been found that it was not able to kill or rupture dormant spores of *Bacillus subtilis*, *Bacillus cereus* or *Bacillus megaterium*, although germinated *Bacillus subtilis* spores were rapidly killed.^225^ This lends insight into the possible limits of mechanical bactericidal approaches.^247^ The efficacy of black silicon surfaces against *Escherichia coli* has also been confirmed, with nanoprotrusion density reported to be more important than length. Interestingly, *Streptococcus gordonii* was unaffected by the surfaces; most likely due to its small size, thicker cell wall and/or lack of motility leading to less lateral movement.^248^ This highlights that antimicrobial effects are dependent on microbe properties, and it is unlikely that any nanocoating will be effective against all microorganisms, all the time. Regarding different properties such as nanoprotrusion height, density and aspect ratio, one study found that three black silicon surfaces with apparently similar nanoarchitecture had different bactericidal efficiencies against different bacteria, though no single variable could be directly correlated with bactericidal efficiency.^249^ This suggests that the variations in properties affecting bactericidal efficiency are subtle, making it difficult to reach a conclusion regarding the best nanotopography for antibacterial properties, and demonstrating that further investigation is needed.

Available data suggest that black silicon may be best suited for use in antimicrobial nanocoatings on unimplanted materials but not *in vivo*, due to its reported ability to rupture mammalian cells (*e.g.*, mouse osteoblasts).^250^ This is in contrast to another study showing that black silicon favored the proliferation of eukaryotic cells (*Cercopithecus aethiops* kidney fibroblast-like cells) without eliciting a host inflammatory response *in vivo* in mice.^251^ Clearly there is need for further research on the biocompatibility of black silicon as it is possible that it may be specific to certain types of eukaryotic cells used, test conditions or specific properties of the surface.

### Nanocomposites

A composite can broadly be defined as a “multicomponent material comprising multiple different phase domains in which at least one phase domain is a continuous phase”,^256^ and these domains are combined to achieve properties not exhibited by any single constituent part.^257^ In the case of nanocomposites, the same definition applies, but at least one of the phases has one dimension at the nanoscale (<100 nm).^256,258^ Generally, antimicrobial nanocomposites tend to take the form of biomaterials with a structural matrix phase, such as a polymer, and antimicrobial NPs (dispersed phase) acting as a filler within that matrix. Thus, biomaterials already in use can be modified to incorporate NPs which confer an antimicrobial effect (Table 4).

**Table 4:** Summary of Additional Selected Examples from the Published Literature Regarding Application of Nanocomposites as Antimicrobial Nanocoatings

Nanocoating description	Aim of application	Key methods	Target organisms	Key results	Source
Dental resin with 70% Bis-GMA and 30% TEGDMA with SiO_2_ and different proportions by weight of MgO NPs	Photocurable dental resin composite with antibacterial properties	Samples seeded with bacterial suspension	*Streptococcus mutans*	Antibacterial activity correlated with proportion of MgO NPs - 1% MgO NP reduced viability by 67.7% and 4% MgO NP by 99.4%	(267)
		Viable counts performed on bacteria liberated from samples into saline after 16–18 h			
A composite dental resin modified by incorporation of TiO_2_ NPs or a TiO_2_/Ag nanocomposite	Antibacterial dental restorative material	Immersion in bacterial suspension for 1 h and further broth added	*Streptococcus mutans*	0.5%, 1% and 2% TiO_2_ and 1% and 2% TiO_2_/Ag NPs significantly inhibited bacterial growth	(268)
		Nonadherent cells enumerated after 18 h		2% TiO_2_ and TiO_2_/Ag NPs significantly reduced biofilm formation	
		Biofilm established by immersion for 7 days			
		Adherent cells enumerated			
ZnO quantum dots incorporated into hydroxyethyl methacrylate and mixed with Bis-GMA	Antimicrobial adhesive resins for application in tooth restoration	Immersion in bacterial suspension	*Streptococcus mutans*	2.16 log reduction in biofilm in treated samples	(269)
		Adherent cells enumerated after 24 h		No cytotoxicity observed	
		Cytotoxicity evaluated against human fibroblasts			
Transbond XT pastes prepared with 1%, 5% and 10% hydroxyapatite/Ag NPs. Resins light-cured for 20 s from each side	Modified orthodontic adhesives to reduce development of caries	Immersion in bacterial suspension	*Lactobacillus acidophilus*	>50% reduction in biofilm formation for all bacteria	(270)
		Adherent cells enumerated after 72 h	*Streptococcus mutans*	Leaching demonstrated, causing dose-dependent loss of bacterial viability	
		Aliquots of leached components inoculated with bacterial	*Streptococcus sanguinis*		
		Viable cells enumerated after 24 h			
Ag NPs and amorphous calcium phosphate NPs incorporated into the Scotchbond multipurpose bonding system	Dental adhesives with antibacterial properties to reduce development of caries	Dental plaque microcosm model	Saliva microbes from healthy donors	Damaged bacteria in treatment group and >50% less live staining vs controls	(271)
		Immersion in saliva combined with broth		Significant reduction of biofilms vs controls	
		Live/dead staining and enumeration of adherent cells after 2 days		50% reduction in cell viability (MTT) and lactic acid production vs controls	
		Metabolic activity (MTT assay) and lactic acid production measured			
Orthodontic composite paste blended with TiO_2_ NPs	Antibacterial orthodontic composites	Immersion in bacterial suspension	*Streptococcus mutans*	>8-fold lower bacterial recovery from experimental specimens versus controls	(272)
		Viable cells enumerated after 48 h		Antibacterial effect lasted 30 days after curing	

An example of a nanocomposite is the incorporation of Ag NPs in poly(lactic-*co*-glycolic acid) (PLGA) grafts, conferring antibacterial properties against an antibiotic-resistant strain of *Staphylococcus aureus* and showing good biocompatibility with MC3T3-E1 preosteoblasts.^259^ This antimicrobial nanocomposite graft was intended for use to improve healing of infected bone defects, and results with infected femoral defects in rats showed greatly improved healing within 12 weeks without evidence of residual bacteria, compared to control grafts which failed to heal in the continued presence of bacteria. Similarly, selenium NPs have been immobilized within PLGA and used to coat bone scaffolds. These materials with a nanocomposite selenium NP-PLGA coating showed antibacterial activity against *Staphylococcus aureus* and *Staphylococcus epidermidis*, and thus offer a potential antibacterial scaffold coating material for use in bone tissue engineering.^260^

The use of composites is common in dentistry due to favorable esthetics and longevity, and their strength and toughness comparable to dental amalgams.^261,262^ This popularity and the relevance of antibacterial activity and tissue integration to dentistry make dental composites ideal candidates for the inclusion of antimicrobial NPs as fillers. A resin-based dental material incorporating a AgBr/cationic polymer nanocomposite was found to have potent bactericidal activity against *Streptococcus mutans*, a relevant oral pathogen, preventing biofilm formation.^263^ Cytotoxicity measured against macrophages was found to be close to that of unmodified resins. Furthermore, the addition of the nanoparticles to the matrix increased the Vickers hardness of the resins, whereas it did not adversely affect their flexural strength. The combination of antimicrobial properties, host biocompatibility and favorable mechanical properties is essential for the development of an effective antimicrobial dental nanocomposite.

Other studies have reported similar success with the modification of dental resins with nanomaterials for improved antibacterial properties and biocompatibility. PMMA has been mixed with modified cellulose nanocrystals decorated with Ag NPs to improve mechanical properties and provide antibacterial activity against *Staphylococcus aureus* and *Escherichia coli,* while causing almost no toxicity to L929 fibroblasts.^264^ PMMA modified with TiO_2_ NPs has also been shown to inhibit the growth of *Candida scotti*,^265^ and PMMA incorporating CuO and TiO_2_ NPs showed significant antimicrobial activity against *Streptococcus salivarius*, *Streptococcus sanguinis*, and *Candida dubliniensis*, while some groups were also active against *Streptococcus mutans*. However, TiO_2_ experimental groups did not show antimicrobial activity against *Streptococcus mutans*.^266^

## Safety of Medicines and Medical Devices Containing Nanocoatings

### The Safety of Patients

The regulatory procedures for approving new nanomedicines and medical devices have been extensively discussed.^273−276^ The pathways and regulations for approving a nanomedicine are generally the same as any other type of medicine. Indeed, the concern from the scientific community is that more nanospecific guidance is needed to smooth the regulatory process along in a safe way that accounts for the novel behaviors and properties of ENMs.^273,275^ Briefly, in the EU, any medicine intended for human use will undergo clinical trials for safety and efficacy according to the Clinical Trials Directive (Directive 2001/20/EC) which sets out the implementation of good clinical practice for such trials, and various codes relating to medicinal products for humans (*e.g.*, Directive 2004/27/EC). Furthermore, regulation EC number 726/2004 indicates the procedure for the authorization and supervision of medicinal products, and this also established the European Medicines Agency (EMA) as an organization with oversight of national level authorities within Europe (Regulation (EC) no. 26/2004). Currently, the EMA offers no overarching guidance on nanospecific issues within those regulations. However, pragmatically, one might also argue that adding to the regulation every time a new type of medicine came along would soon make a cumbersome and unworkable process, and it is for the clinical trial to tease out the substance-specific safety concerns. In the United States, the FDA provides federal regulations on the safety of medicines (*e.g.*, Federal Regulations 21). In 2017, the FDA issued some draft guidance on nanomaterials in drugs and biological products,^277^ but similar to Europe and globally, nanospecific guidance is still being developed. Nonetheless, regardless of geographical location or jurisdiction, the key principles in the safety of medicines should apply. These include demonstrating that the new product is effective for its intended clinical use or more effective than the existing medicine or medical device, and it must be safe for the patient.^273^

In the case of dentistry, in the EU, Annex I of the Medical Devices Directive 93/42/EC traditionally identified requirements on the use of devices that could include dentures and dental implants. This was one part of several pieces of regulation, and for simplicity, these were repealed in 2017 in favor of a more streamlined document (Regulation (EU) 2017/745). The transition period to the new regulation is now complete, and Directive 2017/745 has been mandatory since May 2021. In the UK, following Brexit, the Medicines and Healthcare products Regulatory Agency (MHRA) retained responsibility for medical devices, including those with dental applications under the Medical Devices Regulations 2002^278^ and its amendments, which essentially implements the EU directives. In the United States, the FDA has responsibility for medical devices, and there are a series of steps necessary to bring a product to market, and new devices should be registered with the FDA, undergo premarket safety screening, *etc*. Most of the regulation is detailed in the FDA’s ‘*Title 21 Code of Federal Regulation (CFR), parts 800–1299*’.^279^ Again, all these regulations are intended to be generic and there is no guidance specifically on medical devices containing ENMs, antimicrobials, or other chemical substances. The intended use is a crucial aspect in deciding which regulations need to be followed. So, for example, an ENM coated with an antimicrobial substance might be considered as an antimicrobial drug if it was given systemically or orally, but the same composite material would be considered a medical device if it was part of a dental implant. There are some difficulties in this approach to regulation, for example, where the nanocoating is bioactive and therefore might be both a drug and part of a medical device.

Nonetheless, such regulations ensure that nanosafety can be addressed before a nanomedicine or medical device becomes available for clinical use. Risk is essentially a function of both the type of exposure and the hazard (toxicity), and there are now numerous reviews of the toxicity of ENMs.^107,280−285^ For patients, the route of exposure is defined by the intended treatment method (oral, topical, injection to systemic circulation, *etc*.) and the cumulative dose will be a function of ENM concentration in the medicine, its bioavailability, and the treatment duration. The concern for antimicrobial nanomedicines is that the biocidal component of the material might also be toxic to human cells or tissue. For antimicrobial ENMs made from nutritionally required metals such as zinc or copper, there may be less concern for health because these metals are already in the human body and are homeostatically regulated. However, this is not the case for ENMs made of nonessential toxic metals such as Ag NPs, where the concern is for potential hazard to the internal organs of the patient and/or long-term bioaccumulation. The challenge is to find an effective dose that has the desired antimicrobial effect without causing harm to the surrounding tissue, and in the case of silver, that is certainly possible.^104,286^ The durability of the coating is also a toxicological concern. For example, whether an organic polymer or carbon-based coating could be metabolized to toxic metabolites, but this problem is no different from many traditional medicines that are degraded, and this would be identified in pharmacokinetic studies in Phase 0 clinical trials (animal studies). In other circumstances, it may be desirable for the surface coating to slowly dissolve to release an active biocide (*e.g.*, slow release of silver ions from Ag NPs). This approach is fine provided that the dissolution kinetics of the material are well-defined in the intended tissue or body fluid, thus enabling some understanding of the possible hazard.

For medical devices, the safety concerns are around the use of the device and the effect that may have on the patient. However, for items such as medical instruments or implants, the surgical risks of the operation itself might be similar if the item was nanoenhanced or not. Crucially, the antimicrobial nanocoating on a medical implant would help to minimize the risk of postoperative infection from the wound site, since the biocidal properties of the material would persist after the wound is closed. Similarly, instruments and other devices with antimicrobial coatings would be less likely to introduce infection in the first place. However, the use of surgical disinfectants such as iodine or chlorhexidine should continue since the risk of side effect such as inflammation or dermatitis from the disinfectant is tiny (<1%)^287^ and the relative risks of similar effects from nanocoatings on medical devices will remain unclear until a substantial data set has been collected on use in patients.

In terms of the safety regulations, antimicrobial nanocoatings have some potential advantages over traditional antibiotics. First, there is an assumption that the problem of antibiotic resistance would be less likely to arise from ENMs, and there is evidence that ENMs can tackle antibiotic resistant infections.^288^ Second, nanocoatings have the potential to give persistent antimicrobial activity after surgery, at a time when the postoperative benefits of traditional antibiotics are in doubt.^289^ Finally, many of the antimicrobial nanocoatings can be made of substances that already occur naturally in the human body (zinc, copper) or are part of our diet (*e.g.*, chitosan from shellfish). Thus, there would be less concerns for risk assessment compared to an entirely foreign chemical that is not normally found in the body. However, a quantitative systematic risk analysis comparing antibiotics with ENMs in patient care has not yet been done and the benefits should be weighed against the risks. For example, we may need to be cautious with the use of metallic nanocoatings as antimicrobials because microbes often have genes associated with antibiotic resistance and metal homeostasis on the same plasmid, and there is evidence of metallic ENMs promoting the transfer of antibiotic resistance to other microbes in ecosystems.^290^ There are also theoretical concerns that ENMs in the particulate form may be seen as antigens by the immune system,^291^ although this has not been substantiated in patients, and in any case, adverse effects on immunity or acute inflammation reactions should be detected in early clinical trials before a product comes to market. The schemes that allow clinicians to report the adverse side effects of approved medicines are also not nano-specific. For example, it is not possible to search the MHRA database (‘*yellow card scheme*’)^292^ for “nanomaterials” as all substances are listed by their brand names. Similarly, the *FDA Adverse Event Reporting System* and the ‘*EudraVigilance*’ reporting scheme in the EU both use brand names. In any event, of those nanomedicines approved so far, very few, if any, are based on a coating-mediated effect.^293^

### Occupation Exposure of the Practitioner

In the workplace, safe systems of work are intended to prevent exposure so that there is negligible risk to employees. This approach is used for all new chemicals including ENMs.^294−296^ Potential exposure of the practitioner (*e.g.*, medical doctor, nursing staff, dentist, *etc*.) could arise from incidental inhalation or ingestion of the ENM, or dermal contact. Of course, the usual practice of wearing surgical gloves, a face mask, not eating or drinking while treating patients should minimize these exposure routes. The health concerns for the practitioner would include contact dermatitis caused by handling the novel antimicrobial, the effects of accidental/incidental ingestion or respiratory exposure on health, especially with repeated doses over the working week or longer. These are concerns that apply to all substances in the clinical workplace, but there are some nanospecific issues around setting occupational exposure limits (OELs) for ENMs.^296^ First, uncertainties in the exposure scenario (*e.g.*, exactly how the ENM behaves in aerosols during use, *etc*.) and the bioavailable dose of ENMs have led to the use of a wide range of extrapolation factors, and therefore a broad range of suggested OELs, even for the same material.^296^ Furthermore, most of the OEL studies to date have been on pure ENMs,^296^ and not ENMs applied as coatings, and seemingly not as antibiotic coatings. There are also some specific concerns for the development of antibiotic resistance in the workforce. The latter has been indicated for staff working on the manufacturing of traditional antibiotics,^297^ but the situation is unclear for medical practitioners who would be exposed to much lower quantities in the clinic, or whether novel antimicrobial ENMs (*e.g.*, those made of metals) might present a similar concern for antibiotic resistance in medical staff.

In dentistry, one special concern might be respiratory exposure to the ENMs during dental repairs such as drilling or activities involving abrasion of the tooth. Inevitably, these activities will create an aerosol, but the risks to both practitioners and the patient are yet to be evaluated for ENMs, or ENMs with antimicrobial coatings. Interestingly, with respect to dental prosthetics, the main concern for chemical exposure is during the manufacture and adjustment of the prosthetic, for example, respiratory exposure to ultrafine particles during modeling the shape of the prosthetic with acrylic materials, sandblasting, working with metal alloys, or preparing porcelain veneers.^298^ However, how any hazard quotients or calculation of lifetime cancer risk would be altered by including antimicrobial ENMs in such prosthetics is unknown. Clearly, further research is needed on workplace exposure to ENMs and specifically on antimicrobial ENMs that may have coatings and be made of several chemical substances.

## Concerns Regarding Bacterial Resistance to Antimicrobial Nanomaterials

Although widely reported advantages of antimicrobial nanomaterials are their multiplicity of bacterial targets, and their mechanisms of action which generally differ from those of antibiotics, it is unlikely that they are exempt from the development of bacterial resistance. Where the antimicrobial takes the form of individual agents such as NPs, it is possible for bacteria to develop resistance through sequestration or aggregation of NPs,^299^ efflux,^300,301^ or reduction of ions.^302^ In the case of engineered surfaces with nanotopographical modifications, it is less clear which mechanisms could evolve, though these could incorporate thickening of the cell wall to avoid mechanical disruption, changes to cellular elasticity to reduce rigidity, or changes to surface charge (as in the polymyxin resistance mechanism)^303^ to introduce repellence from the surface to avoid contact with nanostructures.

A particular concern, in the wider context of AMR, is the possibility of the use of non-antibiotic antimicrobials leading to promotion of resistance against antibiotics. This may take the form of co-resistance, where genes conferring resistance to both antibiotics and non-antibiotics are present in the same cell, or cross-resistance, where resistance to a non-antibiotic also results in resistance to an antibiotic (*e.g.*, efflux pumps).^304^ The prevalence of, *e.g.*, silver resistance genes, appears to be low (3.6% in hospital isolates reported), and the presence of resistance genes in the bacterial genome does not necessarily result in phenotypic resistance.^305^ Generally, bacteria more readily develop resistance to antibiotics than to antimicrobial nanoparticles, with resistance to the latter requiring slower, stepwise increases in concentration when investigated *in vitro*.^306^ This suggests that while widespread resistance may not currently be apparent, it is likely to develop eventually with increasing clinical use of antimicrobial nanomaterials.

Multiple studies have reported that certain nanomaterials enhance the transmission of antibiotic resistance genes in *Escherichia coli*, *Staphylococcus aureus*, and *Pseudomonas putida* by transformation and conjugation, two mechanisms of horizontal gene transfer in bacteria. Lu et al.^307^ reported that both Ag NPs and Ag^+^ increase conjugative transfer frequency by inducing ROS overproduction and increasing membrane permeability at environmentally and clinically relevant concentrations. Ding et al.^308^ reported that Al_2_O_3_ NPs, but not bulk Al_2_O_3_, promote plasmid-mediated transformation. This effect was reported to likely be due to Al_2_O_3_ NP-induced damage to the cell membrane allowing plasmids to enter bacteria. This report was later followed up by a finding that certain nanometal oxides (Al_2_O_3_ and ZnO) augment the mutation frequencies of drug-resistant *Escherichia coli* isolates, whereas the corresponding metal ions have weaker effects.^309^ Another study has reported that ZnO and TiO_2_ NPs oppositely impact the transformation efficiency of *Bacillus subtilis*, by modifying the induction of competence; the first step of the transformation process.^310^ The authors showed that two oligopeptide ABC transporters were differentially expressed in response to NPs and thus the effect was due to a physiological adaptation rather than due to cell injury. In contrast, Ag NPs had no significant effect on competence under the same experimental conditions. This was a clear description of NPs in the physiological induction of horizontal gene transfer in bacteria. There are scenarios independent of genetic changes which may also lend a form of resistance to bacteria in a community, for example, *Pseudomonas aeruginosa* produces the metabolite pyocyanin which reduces Ag^+^ to nontoxic Ag^0^; co-incubation experiments have showed increased survival of Ag^+^-exposed bacteria if other pyocyanin-producing bacteria were present as the reduction of local Ag^+^ rendered their environment less toxic.^302^ This is particularly relevant to polymicrobial biofilms where the prevalence of a pyocyanin-producing *Pseudomonas aeruginosa* subset could lend protection to the rest of the biofilm. Another example is β-lactamase-producing bacteria conferring resistance to β-lactam antibiotics to nearby susceptible bacteria due to the excretion of β-lactamase enzymes.^311^ This form of cooperative resistance is independent of genetic modification or the acquisition of resistance elements from other cells.

These studies collectively show that the development of resistance to antimicrobial nanomaterials is inevitable and reinforce the idea that NPs may have the potential to affect dissemination of antibiotic resistance in bacteria, potentially affecting the long-term antibacterial efficacy of nanocoatings as well as posing a public health concern by leading to wider AMR. More generally, this highlights the potential hazards of introducing nanomaterials into the environment without complete understanding of their wider consequences. The vast majority of research conducted on NPs has been conducted in *in vitro* settings and clinical applications will lead to unquantifiable consequences. The extent to which these effects are relevant to nanocoatings needs to be investigated; it has not been demonstrated that nanoparticles presented as surface coatings have a comparable effect regarding competence or induction of horizontal gene transfer. The role of bacterial stress and the potential for nanocoatings to produce subinhibitory or sublethal concentrations of antimicrobials, encouraging more rapid emergence of resistance, needs to be more thoroughly investigated.

## Conclusions

In this review, a range of different nanocoatings have been evaluated. In general, these nanocoatings take two major forms: those carrying active antimicrobial nanoparticles, and those relying on a biomechanical mechanism of action, such as nanoprotrusions. For every introduction of a potential antimicrobial nanocoating, a number of possible new nanocomposite coatings are also produced, allowing the strengths of multiple approaches to be combined. Traditional medical and scientific research has generally favored siloed, individual-disciplined approaches to problems, but serious and impending public health emergencies, such as AMR and its implications for healthcare-acquired infections, require a more multidisciplinary and collaborative approach. The development of antimicrobial nanocoatings is the prototypic example of the interface between microbiology and biomedical engineering. It appears from the evidence synthesized here that antimicrobial nanocoatings will play a significant role in the future protection of surfaces from bacterial colonization, whether those surfaces are environmental or implanted in nature. The benefits of antimicrobial nanocoatings over conventional antibiotics allow targeted effects rather than dispersed and potentially unintended consequences, and combined strategies may be most favorable. A cautious approach should be adopted, ensuring continued biocompatibility, long-term biological activity and minimal ecological impacts, as well as careful consideration of the consequences for bacterial resistance and interactions with AMR. Without doubt, the increasing use of nanomaterials in medicine and dentistry will require the construction of new legislative and regulatory frameworks to ensure safety is maintained and the benefits are maximized. Current evidence suggests an optimistic and exciting future for the use of antimicrobial nanocoatings in improving clinical outcomes in medicine and dentistry.

## Vocabulary

**Engineered nanomaterials**: intentionally manufactured materials containing particles with one or more external dimensions in the size range 1–100 nm
**nanocoating**: a layer or film of particles with one or more external dimensions in the size range 1–100 nm applied to a surface to protect it or improve its function in some capacity
**biofilm**: any consortium of microorganisms in which cells aggregate and become enclosed in a self-produced exopolysaccharide matrix
**antimicrobial resistance**: the outcome of microorganisms changing over time to be able to survive exposure to antimicrobial medicines such as antibiotics which are designed to kill them or inhibit their growth
**surface nanotopography**: the arrangement of physical surface features at the nanoscopic scale
